# A 14-3-3 Family Protein from Wild Soybean (*Glycine Soja*) Regulates ABA Sensitivity in *Arabidopsis*

**DOI:** 10.1371/journal.pone.0146163

**Published:** 2015-12-30

**Authors:** Xiaoli Sun, Mingzhe Sun, Bowei Jia, Chao Chen, Zhiwei Qin, Kejun Yang, Yang Shen, Zhang Meiping, Cong Mingyang, Yanming Zhu

**Affiliations:** 1 Crop Stress Molecular Biology Laboratory, Heilongjiang Bayi Agricultural University, Daqing, P.R. China; 2 Key Laboratory of Agricultural Biological Functional Genes, Northeast Agricultural University, Harbin, P.R. China; National Taiwan University, TAIWAN

## Abstract

It is widely accepted that the 14-3-3 family proteins are key regulators of multiple stress signal transduction cascades. By conducting genome-wide analysis, researchers have identified the soybean 14-3-3 family proteins; however, until now, there is still no direct genetic evidence showing the involvement of soybean 14-3-3s in ABA responses. Hence, in this study, based on the latest *Glycine max* genome on Phytozome v10.3, we initially analyzed the evolutionary relationship, genome organization, gene structure and duplication, and three-dimensional structure of soybean 14-3-3 family proteins systematically. Our results suggested that soybean 14-3-3 family was highly evolutionary conserved and possessed segmental duplication in evolution. Then, based on our previous functional characterization of a *Glycine soja* 14-3-3 protein GsGF14o in drought stress responses, we further investigated the expression characteristics of *GsGF14o* in detail, and demonstrated its positive roles in ABA sensitivity. Quantitative real-time PCR analyses in *Glycine soja* seedlings and GUS activity assays in P_GsGF14O_:GUS transgenic *Arabidopsis* showed that *GsGF14o* expression was moderately and rapidly induced by ABA treatment. As expected, *GsGF14o* overexpression in *Arabidopsis* augmented the ABA inhibition of seed germination and seedling growth, promoted the ABA induced stomata closure, and up-regulated the expression levels of ABA induced genes. Moreover, through yeast two hybrid analyses, we further demonstrated that GsGF14o physically interacted with the AREB/ABF transcription factors in yeast cells. Taken together, results presented in this study strongly suggested that *GsGF14o* played an important role in regulation of ABA sensitivity in *Arabidopsis*.

## Introduction

Abscisic acid (ABA) is a key plant hormone that regulates a wide variety of developmental and physiological processes, including maintenance of seed dormancy [1–3], inhibition of seed germination and seedling growth [4,5], accumulation of free proline [6], control of stomata movement [7,8], regulation of gene expression [9] and plant responses to environmental stress [10]. It has been well demonstrated that ABA participates in plant stress responses through several pathways. Firstly, environmental stress promotes the accumulation of endogenous ABA in leaves [11], and the increased ABA content induces stomata closure in guard cells [12]. Consequently, stomata closure reduces the leaf water loss and protects plant from damages caused by water deficit [13]; at the same time, stomata closure also might depress the gas exchange of plant leaves, decrease the photosynthetic activity and thus lead to growth penalty [12]. Furthermore, ABA also induces the expression of a considerable number of genes, which are always involved in stress responses [14], and promotes the accumulation of free proline [15], which is helpful for osmotic regulation under environmental challenges. Considering the key regulatory roles of ABA in plant development and stress responses, a considerable amount of researches during the past decades have focused on elucidating the ABA signaling transduction pathway.

After years of studies, scientists have established the central ABA signaling transduction pathway, namely the PYR/PYL/RCAR-PP2C-SnRK2 complex mediated signaling cascade [12,16,17]. Under normal conditions, PP2C phosphatases negatively regulate SnRK2 protein kinases by direct interaction and de-phosphorylation of multiple residues within SnRK2s, so the ABA signaling cascade is blocked [18–20]. Upon environmental stress, endogenous ABA accumulates rapidly, and PYR/PYL/RCAR receptors bind ABA, which results in structure change of themselves [21,22]. The ABA-bound PYR/PYL/RCARs then interact with PP2Cs and inhibit PP2C phosphatase activity, and thereby SnRK2s are released and activated [21]. Activated SnRK2s could phosphorylate downstream factors to trigger a series of consequences, such as the membrane ion channels to induce stomata closure [23], or the AREB/ABF transcription factors to regulate ABA induced gene expression [24,25].

The AREB/ABF genes encode the group A subfamily bZIP transcription factors, and nine AREB/ABF homologs have been identified in *Arabidopsis* [9,26,27]. Among them, five AREB/ABFs are well known to be involved in ABA responses, including AREB1/ABF2, AREB2/ABF4, AREB3/ABF1, ABF3, and ABI5. They directly recognize and bind the ABRE cis-elements in the promoter regions of the ABA-responsive genes [26,28]. In addition to SnRK2s, recent studies uncovered that 14-3-3 proteins could also interact with AREB/ABFs and participate in ABA stress responses in plant cells [29–32].

The 14-3-3 family proteins are phosphor-serine/threonine-binding proteins that regulate a wide array of targets via direct protein-protein interaction [33,34]. Up to now, researches have demonstrated the crucial regulatory roles of 14-3-3s in ABA stress responses [35,36]. The 14-3-3s and ABFs interaction has been identified in *Arabidopsis* [36], *Thellungiella* [32] and barley [29]. By conducting genome-wide analysis, previous studies have identified the 14-3-3 family proteins in soybean [37,38]. Among them, SGF14c and SGF14l are found to play critical roles during the early developmental stages of soybean nodules [39]. In addition, SGF14l also could affect isoflavonoid synthesis by regulating the intracellular localization of the GmMYB176 transcription factor [40,41]. In a previous study, we functionally characterized a 14-3-3 family gene from wild soybean (*Glycine soja*), *GsGF14o*, which participated in stomata and root hair development, and negatively regulated plant drought tolerance [37]. However, until now, there is no direct genetic evidence showing the involvement of soybean 14-3-3 proteins in ABA responses.

Hence, in this study, based on the latest *Glycine max* genome on Phytozome v10.3, we initially analyzed the evolutionary relationship, genome organization, gene structure and duplication, and three-dimensional structure of soybean 14-3-3 family members systematically. Then, based on our previous studies [37], we further investigated the expression characteristics of *GsGF14o* in detail, and demonstrated its positive roles in ABA sensitivity. *GsGF14o* was found to be moderately and rapidly induced by ABA stress, and have obvious effect on plant ABA sensitivity, including ABA inhibition on seed germination and seedling growth, as well as ABA induction on stomata closure and gene expression. Finally, according to the protein interaction of GsGF14o with AREB/ABFs in yeasts, we proposed that *GsGF14o* positively regulated plant ABA sensitivity, maybe by directly interacting with AREB/ABF transcription factors.

## Results

### Phylogenetic and Gene Structure Analysis of Soybean 14-3-3 Gene Family

A keyword (14-3-3) search against the soybean (*Glycine max* Wm82.a2.v1) genome at Phytozome v10.3 (http://phytozome.jgi.doe.gov/pz/portal.html) identified a total of twenty sequences containing the 14-3-3 domain (PFAM: PF00244). According to the expression data on Phytozome, two of them (Glyma.20g043700 and Glyma.17G208100) did not express in all detected tissues (S1A Fig). Our previous RNA-seq data of *Glycine soja* roots in response to alkaline stress [42] also showed no expression values for these two genes (S1B Fig). And further check revealed that both of them encoded much fewer amino acids than other soybean 14-3-3 genes, and lacked at least one or more α-helices (nine α-helices for other 14-3-3s). Hence, they are excluded in this study, and detailed information of the remaining eighteen 14-3-3 genes was showed in Table 1.

**Table 1:** Detailed information of soybean 14-3-3 family genes.

Subfamily	Gene Name	locus ID	Location	Alternative Splices	Sequence Length				Position of 14-3-3 Domain
					DNA (bp)	mRNA (bp)	CDS (bp)	Protein (aa)	
non-ε	SGF14g	Glyma.02G208700	Chr02:39388574..39391014 forward	2	2441	1393	789	262	9–245
subfamily	SGF14k	Glyma.14G176900	Chr14:43637893..43642553 forward	2	4661	1377	945	314	61–297
	SGF14i	Glyma.06G101500	Chr06:8052625..8054939 reverse	4	2315	1266	840	279	8–242
	SGF14h	Glyma.04G099900	Chr04:9132954..9135203 reverse	3	2250	1329	867	288	8–242
	SGF14j	Glyma.06G094400	Chr06:7432085..7434388 forward	1	2304	1071	753	250	9–246
	SGF14b	Glyma.04G092600	Chr04:8158031..8160711 forward	1	2681	1344	753	250	9–246
	SGF14a	Glyma.18G298300	Chr18:57587135..57590454 forward	1	3320	1612	774	257	6–242
	SGF14m	Glyma.08G363800	Chr08:47528826..47532060 reverse	3	3235	2054	783	260	6–239
ε subfamily	SGF14r	Glyma.20G025900	Chr20:2845106..2852380 reverse	1	7275	1201	783	260	7–241
	SGF14q	Glyma.07G226000	Chr07:40298318..40302692 reverse	1	4375	1143	780	259	7–241
	SGF14f	Glyma.02G115900	Chr02:11280858..11285331 forward	2	4474	1867	780	259	7–241
	SGF14e	Glyma.01G058000	Chr01:7642485..7646277 forward	1	3793	1358	780	259	7–241
	SGF14p	Glyma.13G270600	Chr13:37265741..37269626 forward	2	3886	1276	792	263	8–242
	SGF14n	Glyma.12G229200	Chr12:38919217..38923409 reverse	2	4193	1227	798	265	13–247
	SGF14d	Glyma.13G290900	Chr13:39120795..39124124 forward	2	3330	1293	786	261	7–240
	SGF14o	Glyma.12G210400	Chr12:36943077..36946491 reverse	2	3415	1370	786	261	7–240
	SGF14l	Glyma.08G115800	Chr08:8877809..8881104 forward	6	3296	1408	780	259	7–241
	SGF14c	Glyma.05G158100	Chr05:35025422..35029392 forward	8	3971	1497	780	259	7–240

**Fig 1:**
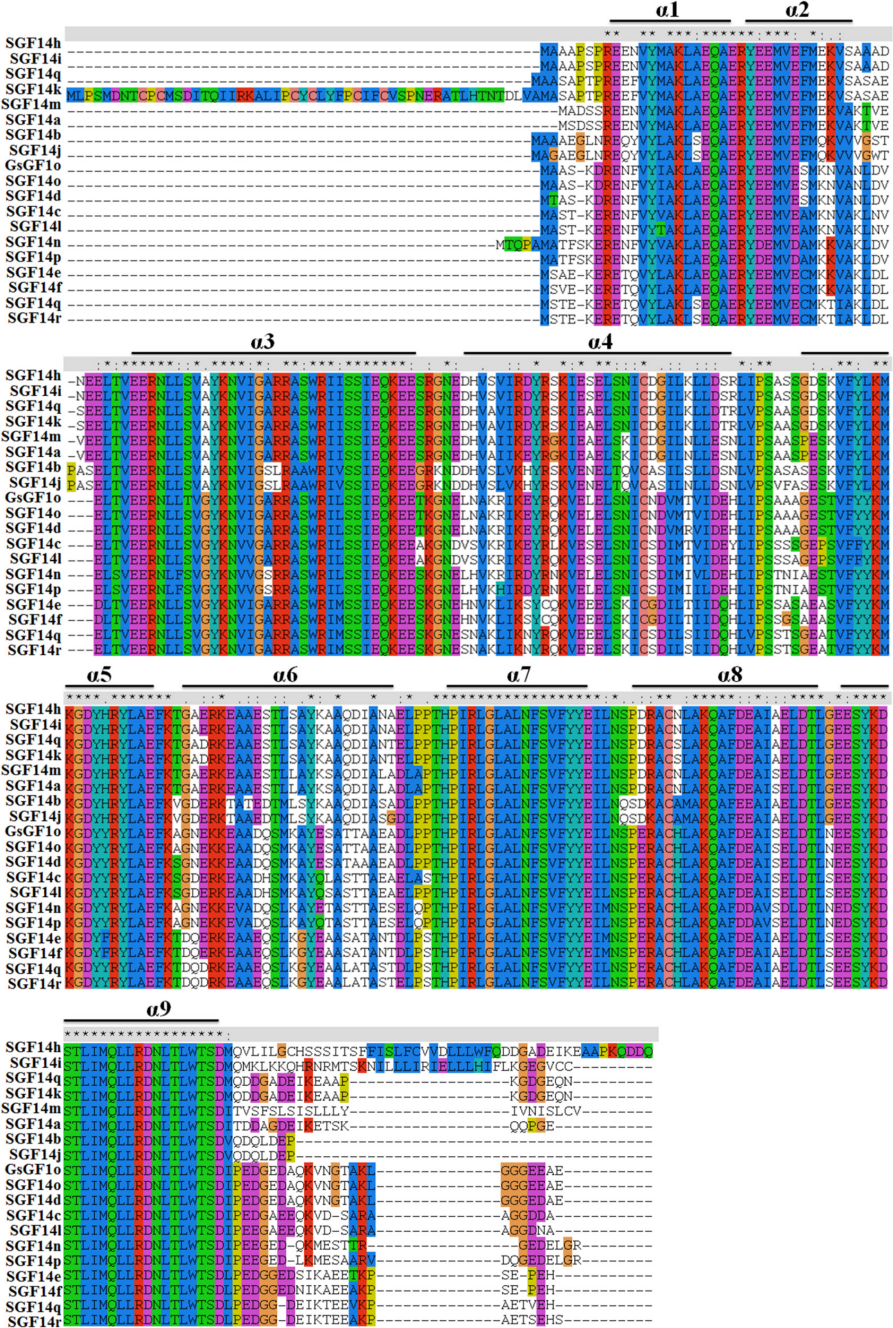
Protein sequence alignment of soybean 14-3-3 family members. Multiple sequences alignment of 14-3-3 proteins was performed by using ClustalX program, and nine α-helices (α1 to α9) were marked with black solid lines.

Previous studies have revealed the conserved structure of 14-3-3 proteins. In this study, we further investigated the protein sequence characteristics and conformational features of soybean 14-3-3 proteins in detail (Fig 1). Protein sequence alignment revealed that similar to 14-3-3s in other species, soybean 14-3-3 proteins were highly conserved in amino acid architecture, and consisted of nine α-helices (α1 to α9). Among them, five α-helices (α1, α3, α5, α7, and α9) were relatively conserved in amino acid sequence than the other four helices, indicating that these five helices might play conserved and important functions during evolution. Notably, the sequences in the C-terminus of soybean 14-3-3s were divergent, and SGF14k possessed an N-terminal extension.

**Fig 2:**
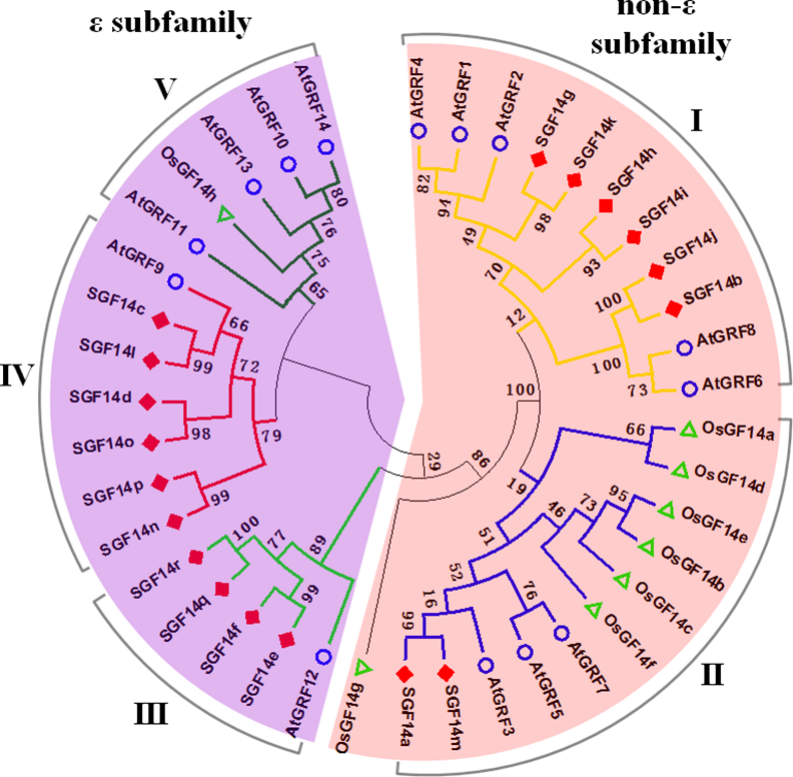
Phylogenetic analysis of the 14-3-3 family proteins from soybean, Arabidopsis and rice. The maximum likelihood phylogenetic tree was constructed by using MEGA5.0 based on the full-length amino acid method with 1000 bootstrap replicates.

**Fig 3:**
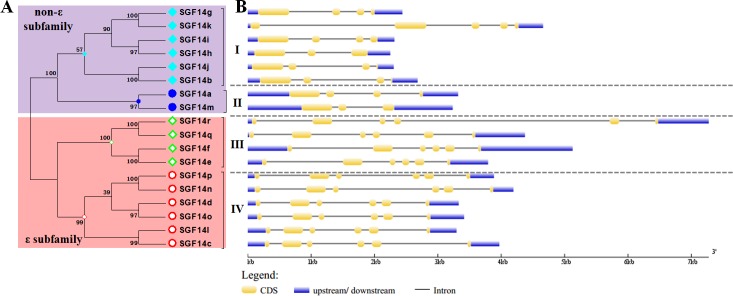
Phylogenetic analysis and exon-intron structures of soybean 14-3-3 family members. (A) The phylogenetic tree of soybean 14-3-3 family proteins. **(**B) The exon-intron structures of soybean 14-3-3 family genes. Yellow and blue boxes represented exons and lines represented introns.

Furthermore, the phylogenetic comparison of the soybean, rice and Arabidopsis 14-3-3s revealed that these 14-3-3 proteins could be clustered into two subfamilies, the ε subfamily and the non-ε subfamily (Fig 2), the same as described previously [38]. Subsequently, the non-ε subfamily was further divided into two groups (Group I-II) and the ε subfamily was divided into three groups (Group III-V). Notably, group I, III and IV only contained soybean and *Arabidopsis* 14-3-3 proteins, while group V only included rice and *Arabidopsis* 14-3-3s. As expected, soybean 14-3-3 family was only consisted of four groups (Group I-IV, Fig 3A), without the fifth group in Fig 2.

Generally speaking, the divergence of exon-intron structure within families always contributes to the evolution of multiple gene families [43]. Paralogous genes within families usually show highly conserved exon-intron organization [44]. Hence, to gain further insights into the structural diversity of soybean 14-3-3 family genes, we then investigated and compared the exon-intron organization in their coding sequences (Fig 3B). As shown in Fig 3B, members from the ε subfamily (group III and IV) averagely shared more introns than the non-ε subfamily (group I and II) members. In details, all of the ε subfamily genes had five introns, while members from the non-ε subfamily averagely possessed three introns (Fig 3B). Furthermore, members within each individual group exhibited similar exon-intron organization pattern, with similar exon numbers and nearly identical exon lengths (Fig 3B). These findings suggested that the exon-intron organization of soybean 14-3-3s was highly conserved within the same group.

### Chromosomal Localization and Segmental Duplication of Soybean 14-3-3 Genes

**Fig 4:**
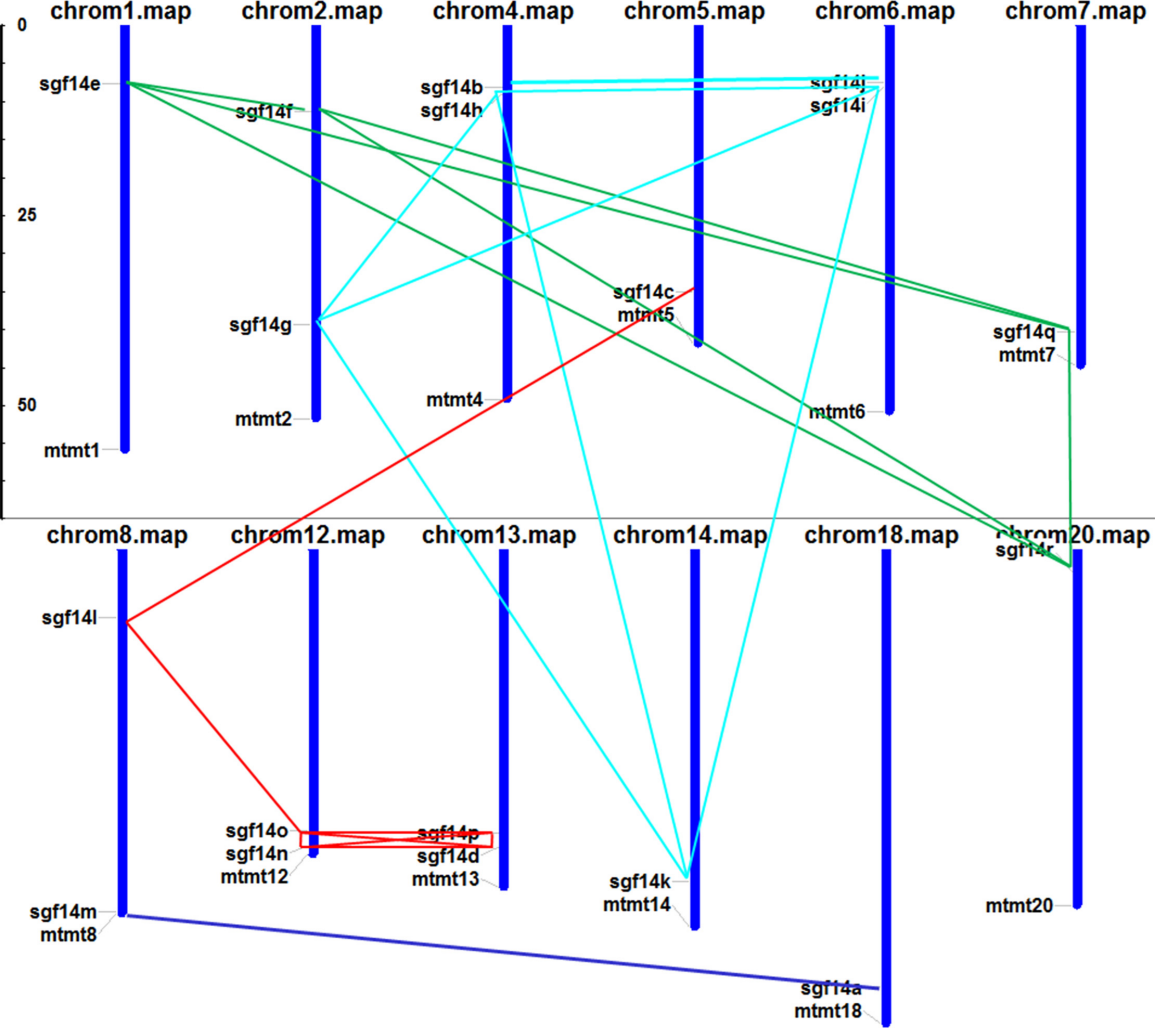
Chromosomal localization and synteny analysis of soybean 14-3-3 family genes. The chromosomal localization of the 14-3-3 family genes was determined by using the MapInspect software. For synteny analysis, the synteny blocks of the soybean genome were downloaded from the Plant Genome Duplication Database (PGDD, http://chibba.agtec.uga.edu/duplication/). Duplicated gene pairs were connected by light blue lines for group I, dark blue lines for group II, green lines for group III, and red lines for group IV.

From the above phylogenetic tree (Fig 2 and Fig 3A), we noticed that all soybean 14-3-3 genes appeared in pairs, indicating possible gene duplication during evolution of the 14-3-3 family. Therefore, we operated chromosome localization and synteny analyses to determine the potential gene duplication within the soybean 14-3-3 family. The chromosomal localization analysis revealed that 18 soybean 14-3-3 genes were distributed among 12 chromosomes (Fig 4). In line with results from phylogenetic tree, synteny analyses further confirmed that the soybean 14-3-3 family did possess gene duplication (Fig 4). Members from group I (linked by light blue lines), group II (linked by dark blue lines), group III (linked by green lines), and group IV (linked by red lines) were found to be located in duplicated blocks, respectively (Fig 4).

**Fig 5:**
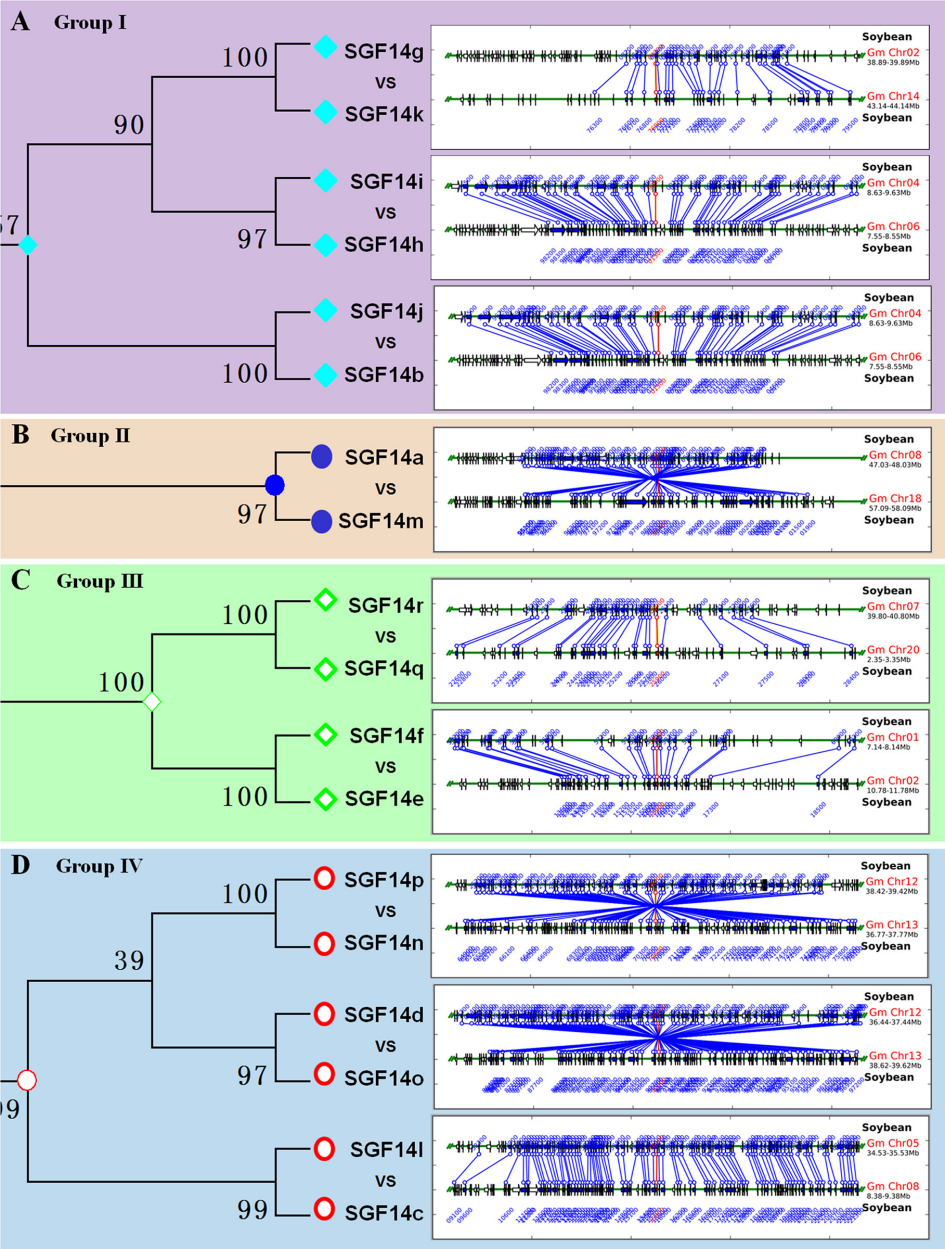
Segmental duplication analyses of soybean 14-3-3 family genes in Group I (A), Group II (B), Group III (C), and Group IV (D). The segmental duplication of soybean 14-3-3 genes was obtained based on the locus search at PGDD website.

It is reported that soybean underwent at least two rounds of genome wide duplications approximately 13 and 59 million years ago [45]. Based on the locus search at PGDD website (Plant Genome Duplication Database, http://chibba.agtec.uga.edu/duplication/)[46], we found that all of the nine 14-3-3 paralogous gene pairs in soybean genome were generated by segmental duplication (Fig 5). Remarkably, four genes in group IV (SGF14o, SGF14p, SGF14n, and SGF14d) were supposed to be within the same duplication blocks (Fig 5D). Moreover, the four 14-3-3 proteins displayed over 80% sequence identity (S2 Fig) and showed closely related location in the phylogenetic tree (Fig 2 and Fig 3A). These findings suggested that these two paralogous gene pairs might originate from a common ancestor, which firstly underwent tandem duplication prior to segmental duplication.

### Structure Analysis of *Glycine soja* GsGF14o Protein

In previous studies, we have isolated a *Glycine soja* 14-3-3 family gene *GsGF14o* [37], which displayed the highest sequence identity to *Glycine max* SGF14o [38], and demonstrated that it negatively regulated plant responses to drought stress. In that research, we showed that GsGF14o shared the highly conserved sequence features with other identified 14-3-3s, including five highly conserved blocks (I-V), two signature motifs (RNLLSVAYKNV and SYKDSTLIMQLLRDNLTLWT), one pseudosubstrate domain for protein kinase C (GARR), one proposed EF-hand domain (SELDTLGEESYKD) and one nuclear exclusion sequence (LIMQLLRDNLTLWT). As shown in Fig 1, GsGF14o also consisted of nine α-helices (α1 to α9).

**Fig 6:**
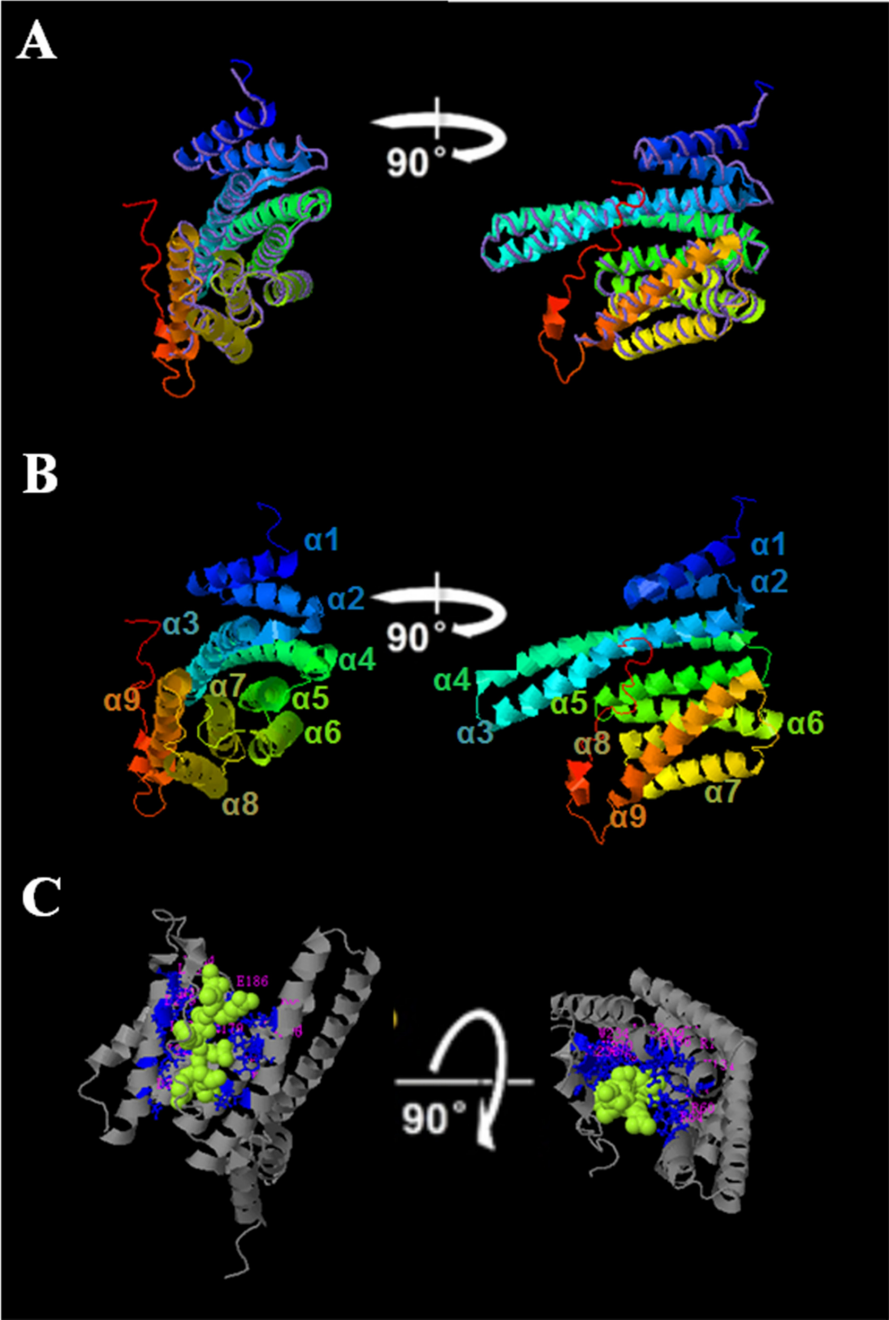
Structural analysis of the *Glycine soja* 14-3-3 protein GsGF14o. (A) Structural comparison of GsGF14o and Nt14-3-3 (PDB: 2098B). GsGF14o structure was shown in cartoon, while Nt14-3-3 structure was shown by using backbone trace. (B) Monomeric structure of the GsGF14o protein. (C) Predicted binding model of GsGF14o. Each structure is rotated 90° to show different view sides of the protein.

To get better understanding of the conformational features of GsGF14o protein, we further predicted the three-dimensional structure of GsGF14o by using the I-TASSER one-line software. As shown in Fig 6A, GsGF14o showed the highest similarity in terms of conformational structure to Nt14-3-3 (PDB: 2o98B) in the Protein Data Bank database (http://www.rcsb.org/pdb/home/home.do). Results of the three-dimensional structure analysis also confirmed that GsGF14o consisted of a bundle of nine α-helices (α1 to α9), which were organized into groups of two (α1 and α2), two (α3 and α4), two (α5 and α6), and three (α7, α8 and α9) helices (Fig 6B). Out of them, the first four helices were reported to be essential for dimer formation [47], and helices α3, α5, α7 and α9 could form a conserved peptide-binding groove (Fig 6C).

### *GsGF14o* Expression Was Induced by ABA Stress

**Fig 7:**
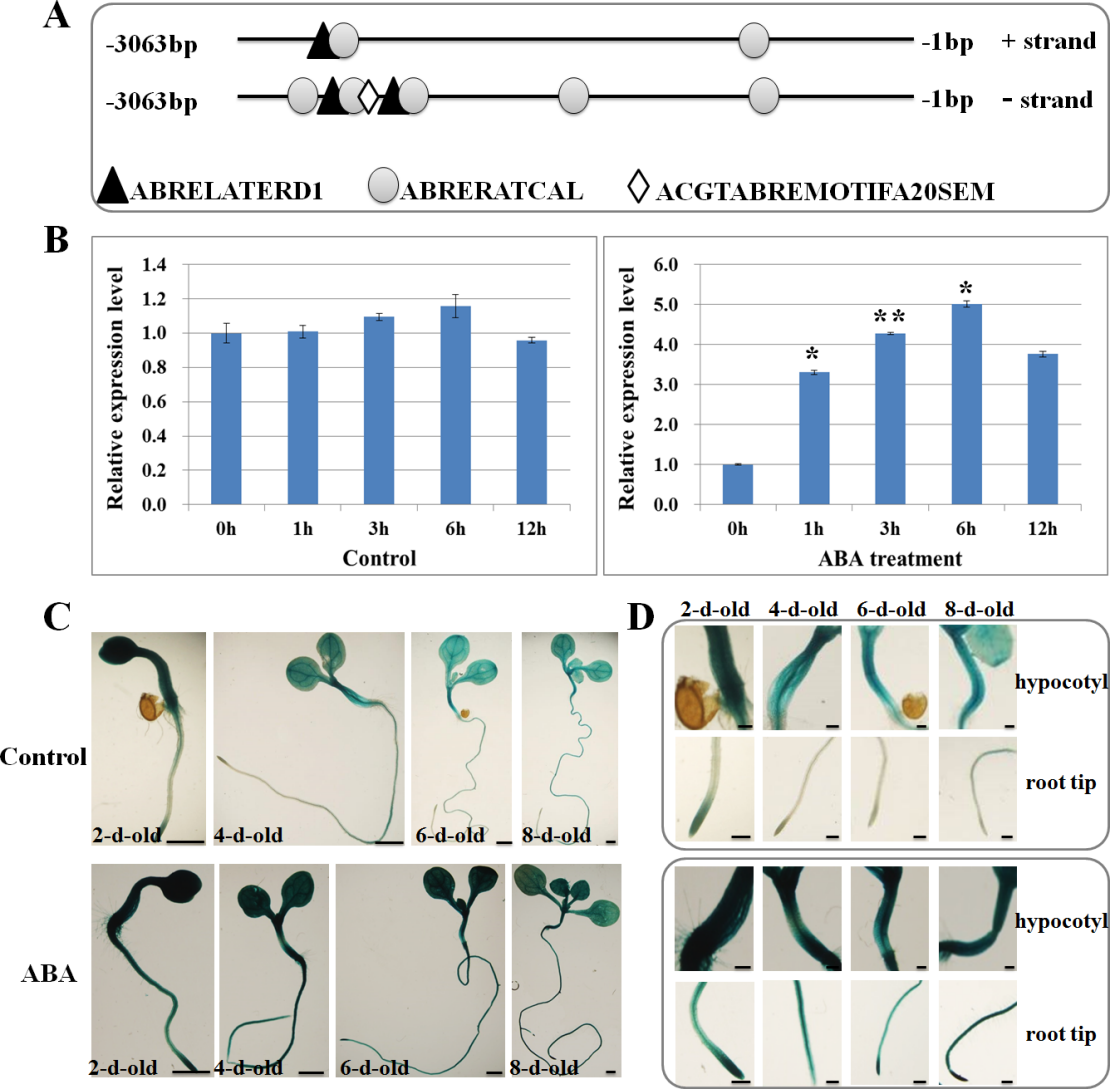
Induced expression of *GsGF14o* in response to ABA stress. (A) The architecture of ABA-responsive cis-elements in *GsGF14o* promoter. (B) Accumulation of *GsGF14o* transcripts in response to ABA stress in wild soybean. *, P < 0.05; **, P < 0.01 by Student’s t-test. (C) Evaluation of GUS expression in P_GsGF14o_:GUS transgenic plants in response to ABA stress. Bars are 1mm. (D) GUS expression in the hypocotyls and root tips of transgenic *Arabidopsis* in response to ABA stress. Bars are 200μm.

In our previous study, we illustrated that the expression levels of *GsGF14o* were greatly and specifically induced by drought stress, but slightly affected by salt and cold stresses [37]. Considering the involvement of 14-3-3s in ABA responses [29,30], we then searched the ABA responsive cis-elements in *GsGF14o* promoter by using PLACE on-line software to check whether *GsGF14o* expression responds to ABA stress. As shown in Fig 7A, we observed several cis-elements related to ABA responses, including three ABRELATERD1 (ACGTG) [48], seven ABRERATCAL (MACGYGB) [49] and one ACGTABREMOTIFA20SEM (ACGTGKC) [50,51] (Fig 7A). The existence of these cis-elements indicated the possible involvement of *GsGF14o* in plant ABA responses.

In this condition, we then investigated the expression profile of *GsGF14o* under ABA stress through quantitative real-time PCR analysis by using RNA extracted from the leaves of 3-week-old *Glycine soja* seedlings treated with 100μM ABA. Our results revealed that without ABA stress, *GsGF14o* displayed stable expression levels (Fig 7B, left), while ABA treatment rapidly increased the transcript accumulation of *GsGF14o* (Fig 7B, right). In details, after ABA treatment, the transcript level of *GsGF14o* started to increase and reached a maximum level at 6 h (about 5 folds), indicating that *GsGF14o* expression responded to ABA stress at the very early stage.

In order to further verify the ABA induction of *GsGF14o* expression, we determined and analyzed the changes in GUS activity in response to ABA stress, by using the P_GsGF14o_:GUS transgenic *Arabidopsis*. To this end, the T_2_ transgenic seedlings grown on normal 1/2MS medium were firstly moved to 1/2MS liquid medium lacking sucrose for hydroponics for 12 h, and then were transferred to 1/2MS medium supplemented with 100μM ABA for ABA stress treatment for 6 h. The GUS staining results showed that the ABA treated seedlings (Fig 7C, ABA) displayed much higher GUS activity than non-treated seedlings (Fig 7C, Control). Specifically speaking, under control condition, the 2-day-old seedlings displayed relatively higher GUS activity, and GUS expression decreased along with the seedling growth (Fig 7C, Control), which is in line with our previous results [37]. However, robust GUS expression was detected in the whole seedlings (2-, 4-, 6-, 8-day-old) treated with ABA (Fig 7C, ABA). Notably, the root tips and hypocotyls of ABA-treated seedlings exhibited much higher GUS activity than that of non-treated plants (Fig 7D). Taken together, these findings strongly suggested that *GsGF14o* expression was indeed induced by ABA stress, and implied potential role of *GsGF14o* in regulating ABA sensitivity.

### *GsGF14o* Overexpression in *Arabidopsis* Augmented the ABA Inhibition on Seed Germination and Seedling Growth

ABA is an important phytohormone that regulates diverse developmental and physiological processes, for example the inhibition of seed germination and seedling growth [10]. To determine the role of *GsGF14o* in ABA responses, we initially checked the seed germination and seedling growth of the wild type (WT) and *GsGF14o* overexpression (OX) *Arabidopsis* lines (line #1, #4, #9) under ABA treatment.

**Fig 8:**
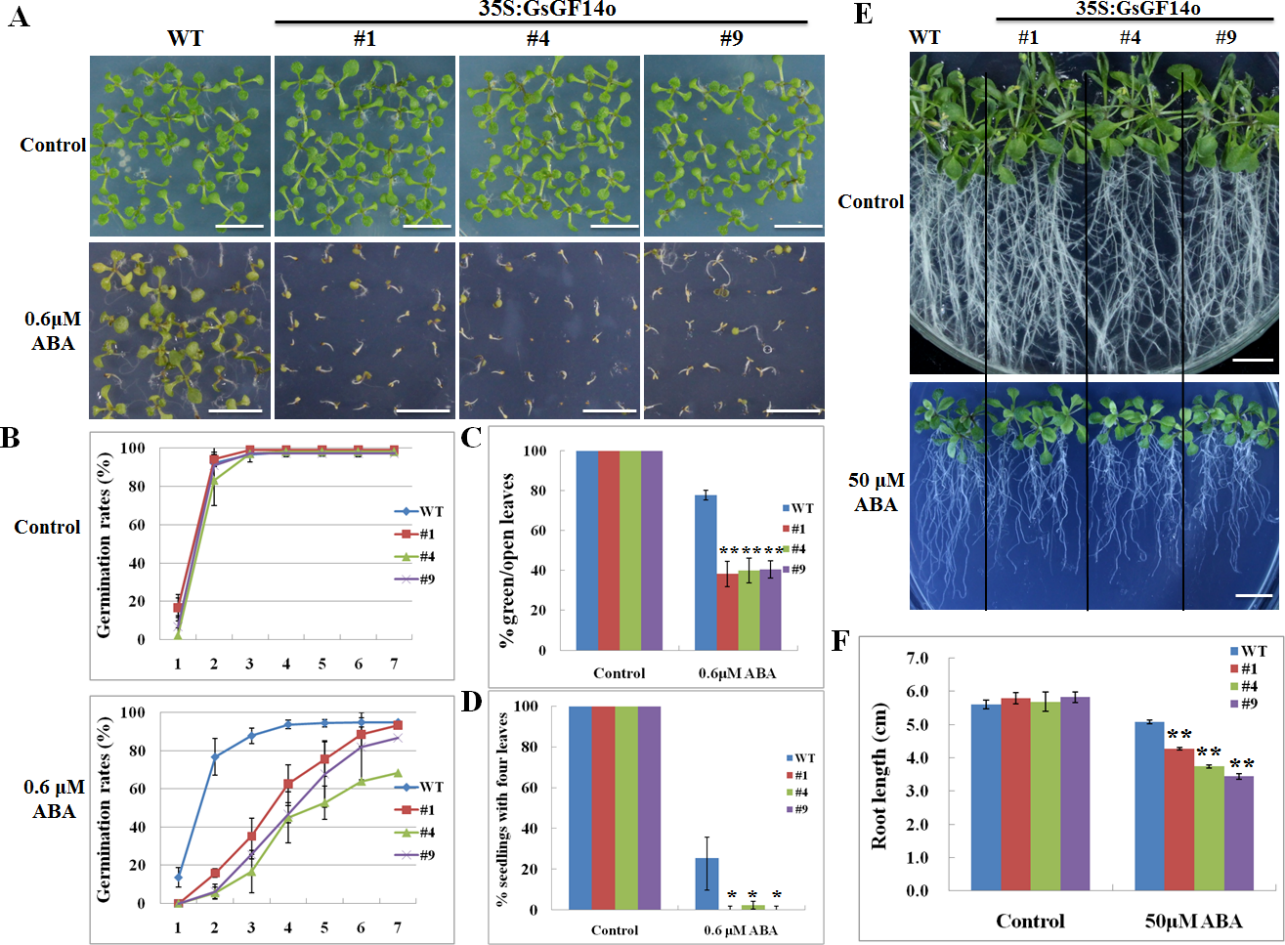
Increased ABA sensitivity of *GsGF14o* transgenic lines during the seed germination and seedling growth stages. (A) The growth performance of WT and *GsGF14o* OX seedlings under ABA stress. Bars are 1cm. (B) Seed germination rates of WT and *GsGF14o* OX lines. (C) Percentage of seedlings with open and green leaves. (D) Percentage of seedlings with four leaves. (E) Phenotypes of WT and OX seedlings. Bars are 1cm. (F) Primary roots of WT and OX seedlings. *, *P* < 0.05; **, *P* < 0.01 by Student’s *t* test.

During the plate seed germination assays, both the WT and OX seeds could germinate and grow well on normal 1/2MS medium (Fig 8A and 8B). However, under 0.6 μM ABA treatment, the WT seeds exhibited much higher germination rates than OX lines (Fig 8B). In details, on the 3^rd^ day after sowing, 87.8% WT seeds could normally germinate, but the germination rates of OX lines were only 35.2% for line 1, 16.7% for line 4, and 25.6% for line 9 (Fig 8B). In addition, ABA application also obviously inhibited the early growth of both WT and OX seedlings (Fig 8A). Consistently, compared with WT, growth of OX seedlings was more severely inhibited by ABA (Fig 8A). Quantification analysis revealed that WT exhibited significantly higher percentages of seedlings with green and open leaves (Fig 8C), and seedlings with four leaves (Fig 8D). In particular, after application of 0.6 μM ABA, almost none of the OX seedlings could develop four leaves, while 25.6% WT seedlings displayed four leaves (Fig 8D). These results suggested that *GsGF14o* OX lines were hypersensitive to ABA stress during the plate seed germination assays.

We further investigated the ABA sensitivity of *GsGF14o* OX lines at the early seedling stage by using the root length assay. To this end, the seven-day-old WT and OX seedlings grown under normal condition were transferred onto 1/2MS medium supplemented with either 0 or 50 μM ABA, and allowed to vertical growth for another 10 days. As shown in Fig 8E, the ABA inhibition on primary root growth of the OX lines was more severe than that of WT (Fig 8E). Statistical analysis also confirmed that the primary roots of the WT seedlings were evidently longer than those of the OX lines under ABA stress (Fig 8F). Taken together, all above results demonstrated that overexpression of *GsGF14o* in *Arabidopsis* resulted in ABA hypersensitivity, and augmented the ABA inhibition effect on seed germination and seedling growth.

### *GsGF14o* Overexpression Promoted ABA Induced Stomata Closure but Not Proline Accumulation

**Fig 9:**
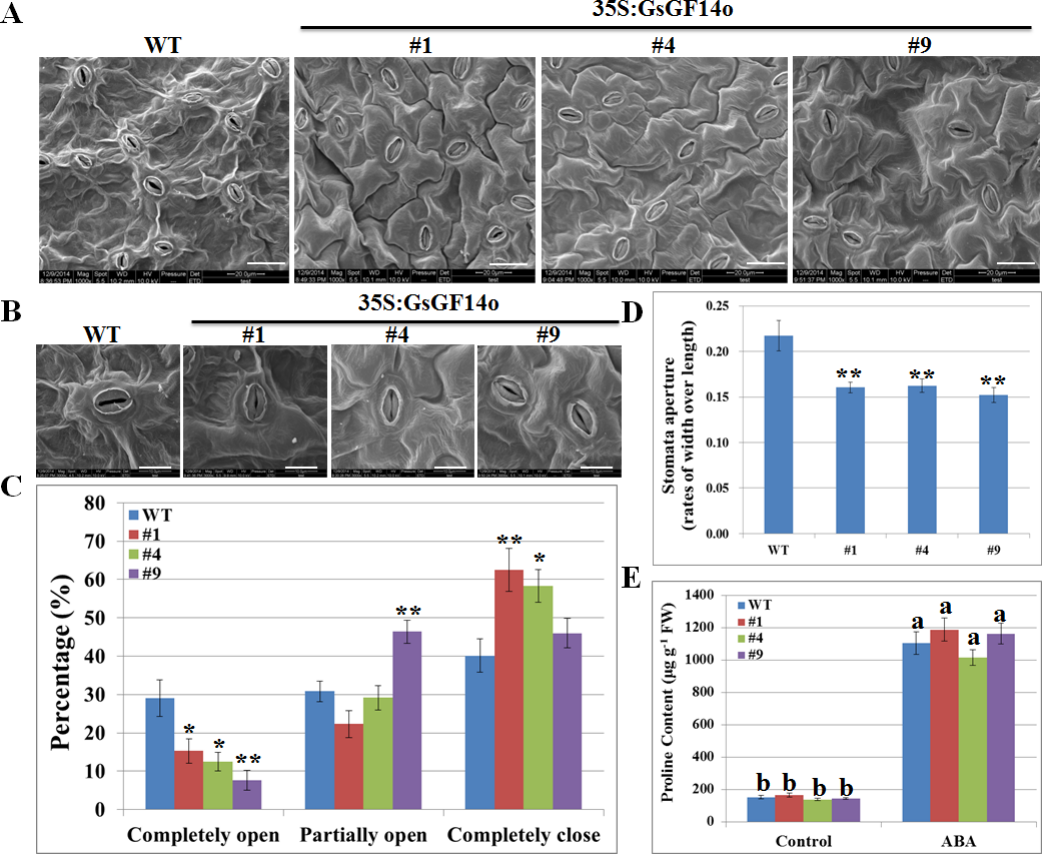
*GsGF14o* overexpression promoted ABA induced stomata closure but not proline accumulation. (A) Stomata from WT and GsGF14o OX lines in response to ABA stress. Bars are 20μm. (B) Presented photos to show stomata from WT and OX lines. Bars are 10μm. (C) Percentage of different stomata in WT and OX lines. (D) Stomata aperture of WT and OX lines. *, *P* < 0.05; **, *P* < 0.01 by Student’s t test. (E) Proline content of WT and OX lines. Different letters indicated statistical differences among means by Duncan's Multiple Range Test (*P* < 0.05).

In addition to the inhibition on seed germination and seedling growth, another two typical effects of ABA on plants are the induced stomata closure and proline accumulation [13]. Our previous study showed that overexpression of *GsGF14o* in *Arabidopsis* resulted in smaller stomata, as evidenced by a decrease of the stomata length and width [37]. In order to test whether *GsGF14o* overexpression affected ABA induced stomata closure, we measured the numbers of completely open, partially open and completely closed stomata (Fig 9), respectively, by using scanning electron microscopy. As shown in Fig 9A and 9B, under ABA treatment, the transgenic lines displayed much less open stomata, but more closed stomata than WT. Statistically speaking, the percentage of completely open stomata was 29% for WT, but these values decreased to 15.2% for line 1, 12.5% for line 4, and 7.9% for line 9. Notably, line 9 displayed higher percentage of partially open stomata than WT, while line 1 and 4 showed more completely closed stomata (Fig 9C). These data illustrated that *GsGF14o* overexpression in *Arabidopsis* promoted ABA induced stomata closure. To confirm this, we further determined stomata apertures (presented by the rates of width over length) of WT and OX lines under ABA treatment. As expected, statistical analysis also suggested that compared with WT, stomata aperture of transgenic lines was more sensitive to ABA (Fig 9D).

In addition to stomata closure, ABA also promotes the accumulation of free proline in plant cells, which may protect plant from adverse environmental challenges. Expectedly, under ABA stress, both WT and OX plants showed a great increase in free proline accumulation. However, beyond our expectation, *GsGF14o* overexpression did not alter the free proline accumulation, as evidenced by similar level of proline content between WT and OX plants under ABA stress (Fig 9E). Taken together, these findings suggested that *GsGF14o* overexpression promoted ABA induced stomata closure, but did not affect ABA induced proline accumulation.

### *GsGF14o* Overexpression Up-Regulated the Expression Levels of ABA Induced Genes

**Fig 10:**
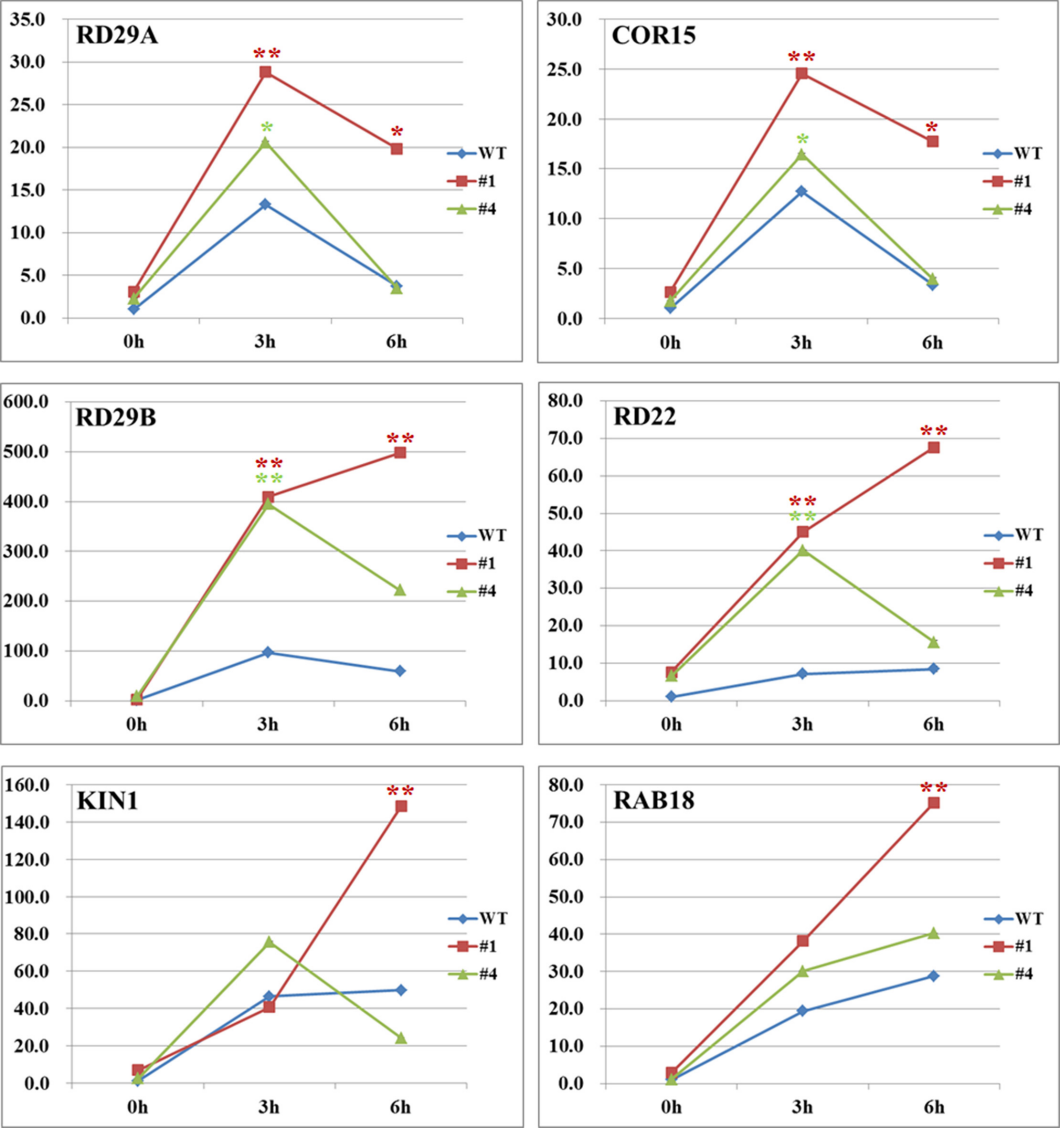
*GsGF14o* overexpression up-regulated the transcript levels of ABA induced genes. Transcription levels of ABA induced genes were determined by quantitative real-time PCR, and *ACTIN2* was used as an internal control. Results were normalized to the corresponding transcript levels of WT plants at 0 h. *, *P* < 0.05; **, *P* < 0.01 by Student’s t test.

ABA stress is suggested to induce the expression of a number of genes, which are involved in plant responses to environmental stress [52]. In this study, we checked the expression of several ABA induced genes, including *RD29A*, *RD29B*, *RD22*, *COR15A*, *KIN1*, and *RAB18*, all of which have ABRE cis-elements in the promoter region. Quantitative real-time PCR results showed that expression levels of all these genes were greatly and rapidly induced by ABA stress in both WT and OX plants. However, their expression levels in OX plants were significantly higher than that in WT (Fig 10). In conclusion, these results suggested that *GsGF14o* overexpression in Arabidopsis up-regulated the expression levels of ABA induced genes.

### GsGF14o Physically Interacted with ABF Transcription Factors in Yeast Cells

**Fig 11:**
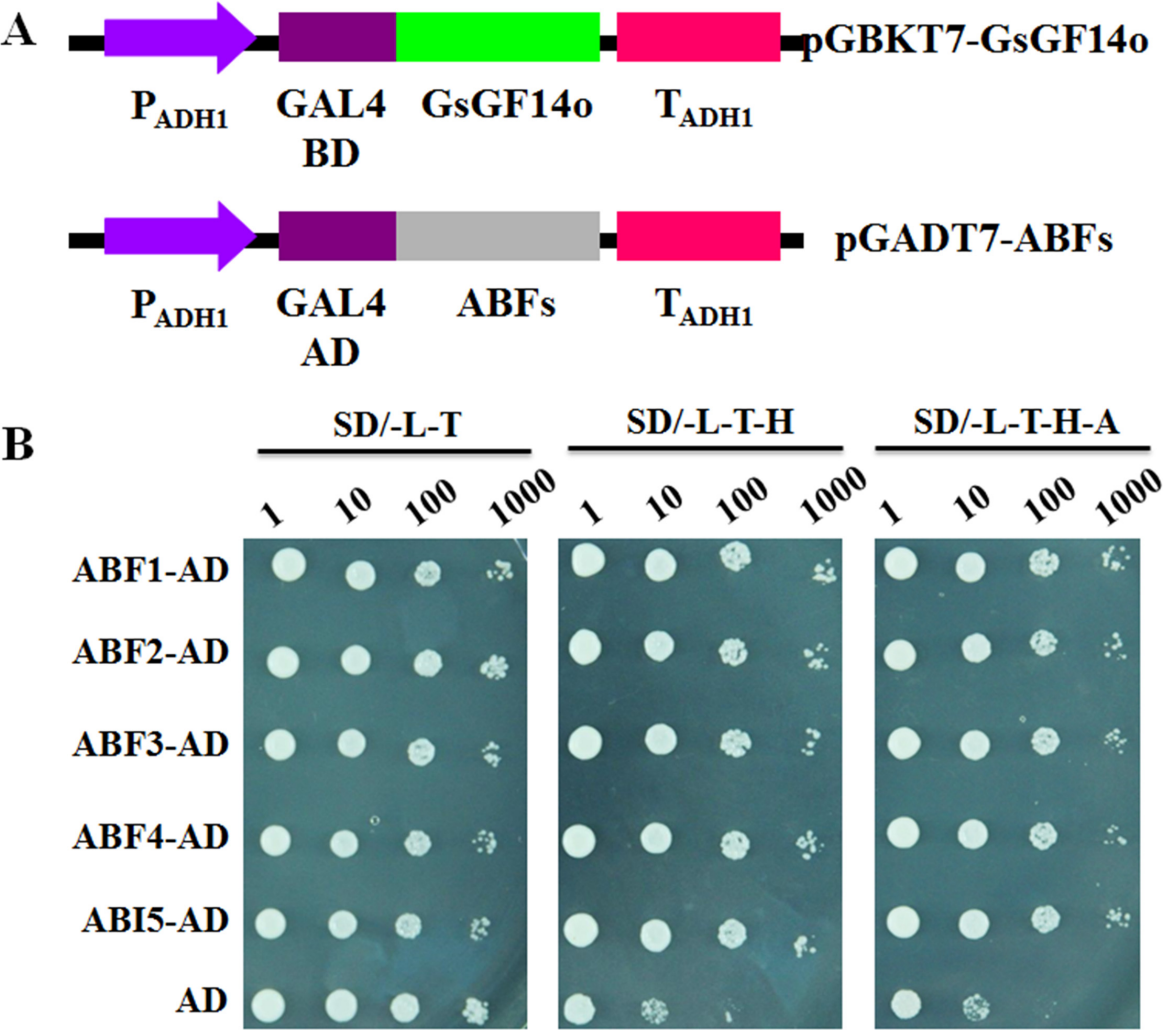
Protein interaction of GsGF14o with ABF transcription factors in yeast cells. (A) Schematic representation of GsGF14o-BD and ABFs-AD fused expression constructors. (B) Yeast two hybrid identification of protein interaction between GsGF14o and ABF transcription factors. The GsGF14o-BD/AD combination was used as a negative control.

Recent studies uncovered that 14-3-3 proteins could physically interact with the AREB/ABF transcription factors, and participate in the ABA signaling transduction in plant cells [29,31,36]. Considering the great changes in expression of these ABRE-containing genes described above (Fig 10), we speculated that GsGF14o might also interact with the AREB/ABF transcription factors. To verify this hypothesis, we used the Y2H (Yeast Two Hybrid) technology to determine whether GsGF14o could interact with AREB/ABFs as described previously [32]. To do this, the *Arabidopsis* ABF1, ABF2, ABF3, ABF4 and ABI5 were in-fused cloned to the pGADT7 vector to express AREB/ABFs at the C-terminus of GAL4 activating domain (Fig 11A). The full-length GsGF14o gene was fused to the GAL4 DNA-binding domain in the pGBKT7 vector, and used as a bait to analyze the protein interaction with AREB/ABFs (Fig 11A). Y2H assays showed that the recombinant yeast cells harboring the pGBKT7-GsGF14o and pGADT7-ABFs vectors could survive well on the SD/-T-L, SD/-T-L-H, and SD/-T-L-H-A selective medium (Fig 11B). However, the yeast cells carrying the pGBKT7-GsGF14o and empty pGADT7 vectors only showed growth on the SD/-T-L medium, but not on the SD/-T-L-H and SD/-T-L-H-A medium. Taken together, these results suggested that GsGF14o did physically interact with AREB/ABFs in yeast cells.

## Discussion

14-3-3 proteins are key regulators of multiple signal transduction cascades relating to diverse physiological and biological processes through hundreds of different protein-protein interactions [53–58]. The development of bioinformatics, especially the whole genome sequencing, dramatically assisted for the genome wide survey of plant 14-3-3 proteins, for example in Arabidopsis [59,60], rice [61,62], tomato [63], cotton [64], and populous [47]. As for soybean, Li et al. identified a total of eighteen 14-3-3 genes based on the soybean genome and EST databases [38]. Here, in this study, we further identified the soybean 14-3-3 family proteins based on *Glycine max* genome at Phytozome v10.3 (Table 1), and suggested that soybean 14-3-3 family was highly evolutionary conserved and possessed segmental duplication in evolution.

It is reported that soybean has undergone at least two rounds of genome wide duplications approximately 13 and 59 million years ago, resulting in large-scale duplicated sequences in soybean genome [45]. Very recently, Tian et al. demonstrated the gene duplication of *Populus* 14-3-3 family, and proposed that purifying selection played a pivotal role in the retention and maintenance of *Populus* 14-3-3 family [47]. Consistently, we also observed evidence for the gene duplication of soybean 14-3-3 family (Figs 2–4). Firstly, almost all soybean 14-3-3 genes appeared in pairs in the phylogenetic tree (Fig 1 and Fig 2A), indicating possible gene duplication during evolution of the 14-3-3 family. Secondly, the chromosomal localization and synteny analyses further confirmed that the soybean 14-3-3 family did possess gene duplication (Fig 3). Lastly but most importantly, we confirmed that nine 14-3-3 paralogous gene pairs in soybean genome were generated by segmental duplication based on the locus search at PGDD website (Fig 4). What is more interesting is that two paralogous pairs in group IV (SGF14d/SGF14o, SGF14n/SGF14p) were supposed to originate from a common ancestor, which firstly underwent tandem duplication prior to segmental duplication.

The 14-3-3 family has been well demonstrated to be highly evolutionary conserved in plants [47,64]. As expected, in this study, we also gave three lines of evidence for the evolutionary conservation of soybean 14-3-3 family. Firstly, previous study reported that the 14-3-3 proteins from 27 species of the Viridiplantae kingdom could be clustered into four groups [65]. Here, we also showed that soybean 14-3-3 family was divided into four groups (Fig 2A). However, when combined with *Arabidopsis* and rice, there was one more group only containing 14-3-3s from Arabidopsis and rice (Fig 1). Secondly, paralogous 14-3-3 genes within the same group showed highly conserved exon-intron organization (Fig 2B), and similar phenomenon was also found in other species, for example *Populus* [47], and *Mulberry Tree* [66]. Thirdly, soybean 14-3-3s, consisting of nine α-helices (α1 to α9), were highly conserved in amino acid architecture (Figs 5 and 6) [37,38].

An increasing body of work has been done to clarify the roles of 14-3-3s in stress response pathways in plants [67]. For example, very recently, 14-3-3s were reported to be positive regulators of primary root growth under control conditions, but be negative regulators in drought stress [68]. Similarly, in a previous study, we also isolated a *Glycine soja* 14-3-3 family protein GsGF14o, and identified it as a negative regulator of plant responses to drought stress [37]. In this study, we further investigated the expression characteristics of *GsGF14o* in detail, and demonstrated its positive roles in ABA sensitivity. Our previous studies reported the expression patterns of *GsGF14o* in different tissues and organs by using GUS staining assays [37]. We found that GUS expression promoted by *GsGF14o* promoter was relatively higher in the 2-day-old seedlings than 4-, 6-, and 8-day-old plants, which was also observed in this study (Fig 7C). Previous study also showed that expression of *GsGF14o* specifically responded to drought stress, as evidenced by a great increase of *GsGF14o* transcript levels under drought stress, but only a slight increase under salt and cold stresses [37]. Here, we further demonstrated that *GsGF14o* expression was also moderately and rapidly induced by ABA treatment through quantitative real-time PCR analysis (Fig 7B) and GUS activity assays (Fig 7C and 7D). The similar phenomenon was also observed for a *Populus* PP2C gene *PeHAB1*, which was markedly induced by drought but moderately induced by ABA [69]. Consistently, the ABA induced expression was also observed for the homologous 14-3-3 genes in other plant species, including *Brassica napus* [70] and *Oryza sativa* [61,62]. These results strongly suggested that *GsGF14o* might be involved in plant ABA responses and signal transduction.

What is interesting is that *GsGF14o* expression predominantly accumulated in root tips and hypocotyls, especially under ABA stress (Fig 7D). The enriched expression of *GsGF14o* in roots further supported its regulatory role in root development reported by our previous studies [37]. Furthermore, consistent with the obvious accumulation of *GsGF14o* in hypocotyls, we also found that *GsGF14o* overexpression led to longer hypocotyls of transgenic *Arabidopsis* seedlings under white, blue and red light (S3 Fig). This observation was in line with previous researches about 14-3-3 involvement in light signaling [71–73]. Correspondingly, previous studies also gave the direct evidence that 14-3-3 proteins regulated the hypocotyl growth [74]. In addition, *Arabidopsis* 14-3-3s were also reported to regulate root growth and chloroplast development as components of the photosensory system [75]. Considering our previous report about the involvement of *GsGF14o* in root hair formation and stomata development, all these findings strongly suggested the multiple regulatory roles of 14-3-3 proteins in plant growth and development.

Even though researches have revealed the responses of 14-3-3 gene expression to ABA stress, little genetic evidence was given to illustrate the biological function of 14-3-3s in ABA responses. In this study, we gave several lines of genetic and molecular evidence showing the regulatory role of *GsGF14o* in ABA sensitivity. Firstly, *GsGF14o* overexpression in *Arabidopsis* augmented the ABA inhibition on seed germination and seedling growth (Fig 8). One important effect of ABA on plants was the inhibition of seed germination and seedling growth [10]. As expected, *GsGF14o* OX lines displayed much lower germination rates, fewer seedlings with open and green leaves and fewer seedlings with four leaves during the plate seed germination assays (Fig 8A–8D), and exhibited shorter primary roots during the root length assays (Fig 8E and 8F). Secondly, overexpression of *GsGF14o* promoted ABA induced stomata closure (Fig 9A–9D), which might help plant deal with the adverse environment. Thirdly, ABA could induce dramatic changes in the transcriptome of plant cells [16,17]. Expectedly, in this study, we elucidated that *GsGF14o* overexpression also increased the expression levels of the ABA responsive genes (Fig 10). Similarly, previous studies also showed that 14-3-3s affected the expression of ABA-responsive genes [29,76]. Taken together, these results strongly suggested *GsGF14o* was a positive regulator of plant ABA responses.

Until now, several researches have indicated the molecular basis of the 14-3-3s involvement in ABA signal transduction [29–32,76]. It is reported that 14-3-3 proteins could directly interact with the AREB/ABF family transcription factors [29,32], which were the key regulators in ABA responses [9,77,78]. For example, in barley, 14-3-3 proteins were found to regulate ABA stress response through HvABI5 interaction [29,30]. In *Thellungiella salsuginea*, the interaction between 14-3-3 proteins and AREB/ABF transcription factors was also reported [32]. However, there is still no report about the protein interaction of soybean 14-3-3 proteins with AREB/ABFs. In the current study, we showed that GsGF14o could physically interact with AREB/ABFs (ABF1, ABF2, ABF3, ABF4 and ABI5) in yeast cells through Y2H analysis (Fig 11). 14-3-3 interaction with targets was reported to be phosphorylation dependent [36,55,79–82]. However, several studies have identified AREB/ABFs interaction in yeast cells for 14-3-3s from soybean, Arabidopsis, barley and *Thellungiella salsuginea*. Since yeast may not have corresponding kinases as in plants, further researches are required to verify the phosphorylation dependence of 14-3-3s and AREB/ABFs interaction. Anyway, AREB/ABFs could then bind the ABRE elements in the promoter regions, and regulate the expression of ABA induced genes, for example *RD29A*, *RD29B*, *RD22*, *COR15A*, *KIN1* and *RAB18* (Fig 10). Hence, we proposed that GsGF14o participated in plant ABA stress responses partly through interacting with AREB/ABFs and regulated the ABA induced genes.

ABA is generally considered as an important hormone, and helps plants to deal with drought stress [83,84]. However, a serial of researches have suggested that an increased ABA sensitivity was not necessarily accompanied with an increased stress tolerance in plants. Plants showing ABA hypersensitivity may also display hypersensitivity to drought stress, in other words, drought stress responses could be controlled by ABA-dependent or ABA-independent pathway. One of the best studied examples is that the ABA overly sensitive mutant *abo3* displayed decreased drought tolerance [85]. Similar results were also found for *Arabidopsis* histone deacetylase gene *AtHD2C* and Lily ABA-, stress-, and ripening-induced gene *LLA23*, overexpression of which decreased plant ABA sensitivity but increased drought tolerance [86,87]. Very recently, transgenic *Arabidopsis* overexpressing a rice group A PP2C gene *OsPP108* was found to be highly insensitive to ABA but tolerant to drought stress [52]. Similarly, our research also revealed that *GsGF14o* overexpression increased ABA sensitivity, but decreased drought tolerance. Remarkably, expression of the ABA inducible gene *RD29A* in *OsPP108* transgenic lines was down-regulated under ABA treatment, but up-regulated under drought stress. Similarly, our results also showed that expression of the ABA inducible stress responsive genes was up-regulated under ABA treatment (Fig 10), but down-regulated under drought stress [37]. Generally, ABA induced stomata closure is an important adaptive response to drought stress, resulting in reduced water loss [88,89]. The *OsPP108* transgenic lines showed ABA insensitivity, with much more open stomata after ABA treatment than WT; in contrast, the water loss of *OsPP108* transgenic lines obviously decreased under drought stress [52]. Opposite phenomenon was also observed for *GsGF14o*. *GsGF14o* transgenic lines displayed ABA hypersensitivity concerning stomata closure (Fig 9), but exhibited decreased drought tolerance, even though with lower water loss rates [37]. The contradiction in ABA sensitivity and drought tolerance could be explained by the hypothesis that this kind of genes might regulate stress tolerance through ABA-independent mechanism.

According to our results, above the ground, *GsGF14o* overexpression reduced the stomata size [37], and promoted ABA-induced stomata closure (Fig 9), which might help plant to reduce the water loss under drought stress [37]. However, at the same time, these changes in stomata size and stomata movement inhibited the gas exchanges, decreased the photosynthetic activity and thus led to growth penalty under drought stress [37]. What is more important, under the ground, *GsGF14o* overexpression led to less and shorter root hairs, as well as shorter primary roots under drought stress [37]. As we know, root hairs enable plants to more effectively extract soil moisture by increasing the effective surface area of roots. In conclusion, these morphological changes of *GsGF14o* transgenic plants finally resulted in reduced drought tolerance. Taken together, it is obvious that *GsGF14o* functions in multiple biological processes, including morphological regulation (stomata and root hair), ABA and drought stress responses.

## Materials and Methods

### Bioinformatic Analysis of the Soybean 14-3-3 Family Genes

To identify the soybean 14-3-3 family genes, a keyword (14-3-3) search against the soybean (*Glycine max* Wm82.a2.v1) genome at Phytozome v10.3 (http://phytozome.jgi.doe.gov/pz/portal.html) was carried out. Multiple sequences alignment of 14-3-3 proteins was performed by using Clustal X, and the maximum-likehood (ML) phylogenetic tree was constructed by using MEGA5.0 with 1000 bootstrap replicates. The exon and intron structures were illustrated by using the Gene Structure Display Server (GSDS, http://gsds.cbi.pku.edu.cn/index.php) [90]. The chromosomal localization of the 14-3-3 family genes was determined by using the MapInspect software. For synteny analysis, the synteny blocks of the soybean genome were downloaded from the Plant Genome Duplication Database (PGDD, http://chibba.agtec.uga.edu/duplication/)[46], and the segmental duplication of soybean 14-3-3 genes was obtained based on the locus search at PGDD website. The three-dimensional structure of GsGF14o protein was predicted by using the I-TASSER software (http://zhanglab.ccmb.med.umich.edu/I-TASSER/)[91]. The cis-elements in *GsGF14o* promoter were predicted by the on-line software PLACE (http://www.dna.affrc.go.jp/PLACE/)[92].

### Plant Material, Growth Conditions and Stress Treatments

Wild soybean seeds (*Glycine soja* 50109) were firstly soaked in 98% sulfuric acid (H_2_SO_4_) for 10 min, washed five times with sterilized distilled water, and then kept in complete darkness with humidity for 2–3 days to promote germination. Germinated seedlings were transferred into 1/4 Hoagland solution and grown at 24–26°C and a 16 h light /8 h dark cycle. For ABA treatment, the roots of 3-week-old seedlings with similar sizes were submerged in 1/4 Hoagland solution supplemented with 100 μM ABA, as described previously [93]. Equal amounts of new-born leaves from 3 individual seedlings were harvested at 0 h, 1 h, 3 h, 6 h and 12 h after ABA application, respectively. Samples were immediately frozen in liquid nitrogen, and then stored at -80°C for RNA extraction.

Seeds of the wild type *Arabidopsis thaliana* (Columbia ecotype) were sterilized with 5% sodium hypochlorite (NaClO) for 6–8 min with shaking, washed with sterilized distilled water for 6–8 times, and then kept at 4°C for 3 days to break seed dormancy. Then *Arabidopsis* were germinated and grown on 1/2MS solid medium or in the standard nutrient solution [94] under controlled environmental conditions (21–23°C, 100 μmol photons m^-2^ s^-1^, 60% relative humidity, 16 h light/8 h dark cycles). To analyze the expression profiles of ABA induced genes, the 3-week-old WT and transgenic *Arabidopsis* seedlings grown in hydroponic were treated with the standard nutrient solution containing 100 μM ABA. Samples were harvested at 0 h, 1 h, 3 h, and 6 h, and prepared for RNA extraction as descried above.

### Quantitative Real-Time PCR Assays

Total RNA was extracted from the 3-week-old wild soybean and/or *Arabidopsis* seedlings by using the EasyPure Plant RNA Kit (Transgen Biotech, China), and then was reversed transcripted to cDNA by using the SuperScript^TM^ III Reverse Transcriptase kit (Invitrogen, Carlsbad, CA, USA). To exclude genomic DNA contamination before quantitative real-time PCR assays, PCR amplification was carried out with specific primers for the internal reference genes, *GADPH* (in *Glycine soja*) and *ACTIN2* (in *Arabidopsis thaliana*). Quantitative real-time PCR assays were performed using a Stratagene MX3000P real-time PCR instrument and the SYBR Select Master Mix (Applied Biosystems, USA). Three independent biological replicates were carried out and subjected to real-time PCR. Gene specific primers are listed in Table 2.

**Table 2:** Gene-specific primers used for quantitative RT-PCR assays.

Gene name	Primer Sequence (5' to 3')
*GsGF14o*	Forward: CTCCAGTCTCTGGGGGATTTG
	Reverse: CTTGTTCCACGTTTTTGCGG
*GAPDH*	Forward: GACTGGTATGGCATTCCGTGT
	Reverse: GCCCTCTGATTCCTCCTTGA
*ACTIN2*	Forward: TTACCCGATGGGCAAGTC
	Reverse: GCTCATACGGTCAGCGATAC
*RD29A*	Forward: GGCGTAACAGGTAAACCTAGAG
	Reverse: TCCGATGTAAACGTCGTCC
*RD29B*	Forward: TGAAGGAGACGCAACAAGGG
	Reverse: CAACGGTGGTGCCAAGTGAT
*COR15*	Forward: AATTTCAAGCACTTAAACTCGT
	Reverse: AGAATGTGACGGTGACTGTG
*KIN1*	Forward: AACAAGAATGCCTTCCAAGC
	Reverse: CGCATCCGATACACTCTTTCC
*RD22*	Forward: GGTTCGGAAGAAGCGGAG
	Reverse: GAAACAGCCCTGACGTGATAT
*RAB18*	Forward: CTTGGGAGGAATGCTTCAC
	Reverse: CTTCTTCTCGTGGTGCTCAC

### GUS Histochemical Staining

To explore the GUS expression in the P_GsGF14o_:GUS transgenic *Arabidopsis* in response to ABA, seeds of the T_2_ generation transgenic lines (two independent lines) were sowed and grown on normal 1/2MS medium for 2, 4, 6 and/or 8 days, respectively. The young seedlings were moved to 1/2MS liquid medium lacking sucrose for hydroponics for 12 h, and were then transferred to 1/2MS medium supplemented with 100 μM ABA for ABA stress treatment for 6 h. Three or four seedlings from each line and each condition were sampled and subjected to GUS staining. GUS staining was performed by using 5-bromo-4-chloro-3-indolyl-b-D-glucuronide (X-Gluc) as substrate [95].

### Seed Germination and Root Length Assays in Response to ABA Stress

In the plate germination assays, WT and OX *Arabidopsis* seeds were germinated and grown on normal 1/2MS medium or 1/2MS medium supplemented with 0.6 μM ABA. The germination rates were recorded for consecutive 7 days after sowing. Pictures were taken on the 7^th^ day to show the growth performance of each line. The numbers of seedlings with open and green leaves and/or seedlings with four rosette leaves were recorded. Ninety seeds of each line were used for each experiment and the experiments were repeated three times.

In the root length assays, the 7-day-old WT and OX seedlings grown on normal 1/2MS medium were transferred to fresh medium without or with 50 μM ABA, and grown vertically for another 10 days. The primary root length of each seedling was measured and recorded. Fifteen seedlings of each line were used for each experiment and the experiments were repeated three times.

### Measurement of the Stomata Aperture

To measure the ABA induced stomata closure, rosette leaves of the 3-week-old WT and OX seedlings were detached and floated (abaxial side down) on the solution containing 30 mM KCl, 0.1 mM EGTA, and 10 mM MES-KOH (pH 6.15) under light for 2.5 h, to induce stomatal opening. Then, leaves were transferred to solution containing 30 mM KCl, 0.1 mM CaCl_2_, 10 mM MES-KOH (pH 6.15), and 10 μM ABA for 2 hours. After that, leaves were fixed in the 0.1 M phosphate buffer (pH 7.4) containing 2.5% glutaraldehyde and 4% para-formaldehyde at 4°C for 3 days, mounted onto standard aluminum stubs for the Hitachi scanning electron microscope, and then sputter-coated with approximately 30 nm gold using a sputter coater (K550, Emitech). The images were then viewed using an S2400 scanning electron microscope (Hitachi) with an accelerating voltage of 1.5 kV. For statistical analysis, 30 stomata were used for each line, and the ratio of length over width was used to represent stomata aperture.

### Measurement of Free Proline Content

To analyze the changes of free proline accumulation in response to ABA stress, the 10-day-old WT and OX *Arabidopsis* seedlings grown on normal 1/2MS medium were transferred onto 1/2MS medium with 50 μM ABA for 4 days. Proline content was measured by using the ninhydrin assay as described [96]. Briefly, approximately 0.5 g fresh harvested leaves were homogenized in 3% sulfosalicylic acid, and reacted with acid-ninhdrin and glacial acetic acid for 1 hour at 100°C. The reaction mixture was extracted with toluene, and free proline concentration was calculated by using the values of the absorbance at 520 nm.

### Yeast Two Hybrid Assays

The full-length coding region of *GsGF14o* was amplified by using the following primer pairs: 5’-TAAGAATTCATGGCTGCCTCCAA-3’ and 5’-TTTGTCGACCACTCTGCCTCCTC-3’. The PCR products were cloned to the pGBKT7 vector to express the GsGF14o-BD fused protein. The full-length coding regions of Arabidopsis ABF transcription factors were amplified by using the following primer pairs: 5’-TCCATCGATACATGGGTACTCAC ATTGAT-3’ and 5’-TATCTCGAGACCTTCTTACCACGGACC-3’ (for ABF1), 5’-TCCATCGATACATGGATGGT AGTATGAATTTG-3’ and 5’-TATCTCGAGACCAAGGTCCCGACTCTGT-3’ (for ABF2), 5’-TCCATCGATACA TGGGGTCTAGATTAAACTTC-3’ and 5’-TATCTCGAGACCAGGGACCCGTCAAT-3’ (for ABF3), 5’-TCCATC GATACATGGGAACTCACATCAAT-3’ and 5’-TATCTCGAGACCATGGTCCGGTTAATGT-3’ (for ABF4), 5’-TCCATCGATACATGGTAACTAGAGAAACGAAG-3’ and 5’-TATCTCGAGAGAGTGGACAACTCGGGTT-3’ (for ABI5). The PCR products were cloned to the pGADT7 vector to express the ABFs-AD fused proteins.

To perform the yeast two hybrid analysis, the pGBKT7-GsGF14o and pGADT7-ABFs constructs were introduced into the yeast strain AH109 using the lithium acetate method, and the culture (OD600 = 0.6) of the PCR-positive transformants were serial diluted (1:10, 1:100, 1:1000), and then pointed onto SD/-Trp-Leu, SD/-Trp-Leu-His, and SD/-Trp-Leu-His-Ade medium.

## Supporting Information

S1 Fig Expression patterns of the soybean 14-3-3 family genes. (A) Expression profiles of soybean 14-3-3s in different tissues. (B) Expression profiles of soybean 14-3-3s in response to alkaline stress (50 mM NaHCO_3_, pH 8.5) based on the RNA-seq data.
(TIF)

S2 Fig Sequence identity and alignment of four group I 14-3-3 proteins. (A) Sequence identity among the four group I 14-3-3 proteins. (B) Multiple sequence alignment of the four group I 14-3-3 proteins.
(TIF)

S3 Fig *GsGF14o* overexpression led to longer hypocotyls of transgenic *Arabidopsis* seedlings. (A) Representative photos to show the hypocotyls from WT and *GsGF14o* OX seedlings under white, blue and red light. (B) Comparison of the hypocotyl length of WT and OX lines.
(TIF)

## References

[1] Gonzalez-Garcia MP, Rodriguez D, Nicolas C, Rodriguez PL, Nicolas G, Lorenzo O. Negative regulation of abscisic acid signaling by the *Fagus sylvatica* FsPP2C1 plays a role in seed dormancy regulation and promotion of seed germination. Plant Physiol 2003; 133: 135–144. 12970481 10.1104/pp.103.025569 PMC196589

[2] Nambara E, Marion-Poll A. ABA action and interactions in seeds. Trends Plant Sci 2003; 8: 213–217. 12758038 10.1016/S1360-1385(03)00060-8

[3] Finkelstein R, Reeves W, Ariizumi T, Steber C. Molecular aspects of seed dormancy. Annu Rev Plant Biol 2008; 59: 387–415. 10.1146/annurev.arplant.59.032607.092740 18257711

[4] Finkelstein RR, Gampala SS, Rock CD. Abscisic acid signaling in seeds and seedlings. Plant Cell 2002; 14 Suppl: S15–45. 12045268 10.1105/tpc.010441 PMC151246

[5] Arc E, Sechet J, Corbineau F, Rajjou L, Marion-Poll A. ABA crosstalk with ethylene and nitric oxide in seed dormancy and germination. Front Plant Sci 2013; 4: 63 10.3389/fpls.2013.00063 23531630 PMC3607800

[6] Sharma S, Verslues PE. Mechanisms independent of abscisic acid (ABA) or proline feedback have a predominant role in transcriptional regulation of proline metabolism during low water potential and stress recovery. Plant Cell Environ 2010; 33: 1838–1851. 10.1111/j.1365-3040.2010.02188.x 20545884

[7] Wang Y, Chen ZH, Zhang B, Hills A, Blatt MR. PYR/PYL/RCAR abscisic acid receptors regulate K^+^ and Cl^-^ channels through reactive oxygen species-mediated activation of Ca^2+^ channels at the plasma membrane of intact Arabidopsis guard cells. Plant Physiol 2013; 163: 566–577. 10.1104/pp.113.219758 23899646 PMC3793038

[8] Okamoto M, Peterson FC, Defries A, Park SY, Endo A, Nambara E, et al Activation of dimeric ABA receptors elicits guard cell closure, ABA-regulated gene expression, and drought tolerance. P Natl Acad Sci USA 2013; 110: 12132–12137. 10.1073/pnas.1305919110 PMC3718107 23818638

[9] Yoshida T, Fujita Y, Maruyama K, Mogami J, Todaka D, Shinozaki K, et al Four Arabidopsis AREB/ABF transcription factors function predominantly in gene expression downstream of SnRK2 kinases in abscisic acid signalling in response to osmotic stress. Plant Cell Environ 2015; 38: 35–49. 10.1111/pce.12351 24738645 PMC4302978

[10] Fujii H, Verslues PE, Zhu JK. Identification of two protein kinases required for abscisic acid regulation of seed germination, root growth, and gene expression in Arabidopsis. Plant Cell 2007; 19: 485–494. 17307925 10.1105/tpc.106.048538 PMC1867333

[11] Finkelstein R. Abscisic Acid synthesis and response. The Arabidopsis book / American Society of Plant Biologists 2013; 11: e0166 10.1199/tab.0166 24273463 PMC3833200

[12] Osakabe Y, Yamaguchi-Shinozaki K, Shinozaki K, Tran LS. ABA control of plant macroelement membrane transport systems in response to water deficit and high salinity. New Phytol 2014; 202: 35–49. 10.1111/nph.12613 24283512

[13] Ou X, Gan Y, Chen P, Qiu M, Jiang K, Wang G. Stomata prioritize their responses to multiple biotic and abiotic signal inputs. PLoS ONE 2014; 9: e101587 10.1371/journal.pone.0101587 25003527 PMC4086820

[14] Lim S, Baek W, Lee SC. Identification and functional roles of CaDIN1 in abscisic acid signaling and drought sensitivity. Plant Mol Biol 2014; 86: 513–525. 10.1007/s11103-014-0242-5 25149469

[15] De Ollas C, Arbona V, Gomez-Cadenas A. Jasmonic acid interacts with abscisic acid to regulate plant responses to water stress conditions. Plant Signal Behav 2015; In press. 10.1080/15592324.2015.1078953 PMC4854360 26340066

[16] Umezawa T, Nakashima K, Miyakawa T, Kuromori T, Tanokura M, Shinozaki K, et al Molecular basis of the core regulatory network in ABA responses: sensing, signaling and transport. Plant Cell Physiol 2010; 51: 1821–1839. 10.1093/pcp/pcq156 20980270 PMC2978318

[17] Raghavendra AS, Gonugunta VK, Christmann A, Grill E. ABA perception and signalling. Trends Plant Sci 2010; 15: 395–401. 10.1016/j.tplants.2010.04.006 20493758

[18] Fujii H, Zhu JK. Arabidopsis mutant deficient in 3 abscisic acid-activated protein kinases reveals critical roles in growth, reproduction, and stress. P Natl Acad Sci USA 2009; 106: 8380–8385. 10.1073/pnas.0903144106 PMC2688869 19420218

[19] Fujita Y, Nakashima K, Yoshida T, Katagiri T, Kidokoro S, Kanamori N, et al Three SnRK2 protein kinases are the main positive regulators of abscisic acid signaling in response to water stress in Arabidopsis. Plant Cell Physiol 2009; 50: 2123–2132. 10.1093/pcp/pcp147 19880399

[20] Leung J, Merlot S, Giraudat J. The Arabidopsis ABSCISIC ACID-INSENSITIVE2 (ABI2) and ABI1 genes encode homologous protein phosphatases 2C involved in abscisic acid signal transduction. Plant Cell 1997; 9: 759–771. 9165752 10.1105/tpc.9.5.759 PMC156954

[21] Ma Y, Szostkiewicz I, Korte A, Moes D, Yang Y, Christmann A, et al Regulators of PP2C phosphatase activity function as abscisic acid sensors. Science 2009; 324: 1064–1068. 10.1126/science.1172408 19407143

[22] Nishimura N, Hitomi K, Arvai AS, Rambo RP, Hitomi C, Cutler SR, et al Structural mechanism of abscisic acid binding and signaling by dimeric PYR1. Science 2009; 326: 1373–1379. 10.1126/science.1181829 19933100 PMC2835493

[23] Yoshida R, Umezawa T, Mizoguchi T, Takahashi S, Takahashi F, Shinozaki K. The regulatory domain of SRK2E/OST1/SnRK2.6 interacts with ABI1 and integrates abscisic acid (ABA) and osmotic stress signals controlling stomatal closure in Arabidopsis. J Biol Chem 2006; 281: 5310–5318. 16365038 10.1074/jbc.M509820200

[24] Kobayashi Y, Murata M, Minami H, Yamamoto S, Kagaya Y, Hobo T, et al Abscisic acid-activated SNRK2 protein kinases function in the gene-regulation pathway of ABA signal transduction by phosphorylating ABA response element-binding factors. Plant J 2005; 44: 939–949. 16359387 10.1111/j.1365-313X.2005.02583.x

[25] Nakashima K, Fujita Y, Kanamori N, Katagiri T, Umezawa T, Kidokoro S, et al Three Arabidopsis SnRK2 protein kinases, SRK2D/SnRK2.2, SRK2E/SnRK2.6/OST1 and SRK2I/SnRK2.3, involved in ABA signaling are essential for the control of seed development and dormancy. Plant Cell Physiol 2009; 50: 1345–1363. 10.1093/pcp/pcp083 19541597

[26] Yang X, Yang YN, Xue LJ, Zou MJ, Liu JY, Chen F, et al Rice ABI5-Like1 regulates abscisic acid and auxin responses by affecting the expression of ABRE-containing genes. Plant Physiol 2011; 156: 1397–1409. 10.1104/pp.111.173427 21546455 PMC3135944

[27] Lopez-Molina L, Mongrand S, McLachlin DT, Chait BT, Chua NH. ABI5 acts downstream of ABI3 to execute an ABA-dependent growth arrest during germination. Plant J 2002; 32: 317–328. 12410810 10.1046/j.1365-313x.2002.01430.x

[28] Xu D, Li J, Gangappa SN, Hettiarachchi C, Lin F, Andersson MX, et al Convergence of Light and ABA signaling on the ABI5 promoter. PLoS Genet 2014; 10: e1004197 10.1371/journal.pgen.1004197 24586210 PMC3937224

[29] Schoonheim PJ, Costa Pereira DD, De Boer AH. Dual role for 14-3-3 proteins and ABF transcription factors in gibberellic acid and abscisic acid signalling in barley (*Hordeum vulgare*) aleurone cells. Plant Cell Environ 2009; 32: 439–447. 10.1111/j.1365-3040.2009.01932.x 19143991

[30] Schoonheim PJ, Sinnige MP, Casaretto JA, Veiga H, Bunney TD, Quatrano RS, et al 14-3-3 adaptor proteins are intermediates in ABA signal transduction during barley seed germination. Plant J 2007; 49: 289–301. 17241451 10.1111/j.1365-313X.2006.02955.x

[31] Schoonheim PJ, Veiga H, Pereira Dda C, Friso G, van Wijk KJ, de Boer AH. A comprehensive analysis of the 14-3-3 interactome in barley leaves using a complementary proteomics and two-hybrid approach. Plant Physiol 2007; 143: 670–683. 17172288 10.1104/pp.106.090159 PMC1803744

[32] Vysotskii DA, de Vries-van Leeuwen IJ, Souer E, Babakov AV, de Boer AH. ABF transcription factors of Thellungiella salsuginea: Structure, expression profiles and interaction with 14-3-3 regulatory proteins. Plant Signal Behav 2013; 8: e22672 10.4161/psb.22672 23221757 PMC3745569

[33] Roberts MR. 14-3-3 Proteins find new partners in plant cell signalling. Trends Plant Sci 2003; 8: 218–223. 12758039 10.1016/S1360-1385(03)00056-6

[34] Ferl RJ. 14-3-3 proteins: regulation of signal-induced events. Physiol Plantarum 2004; 120: 173–178. 10.1111/j.0031-9317.2004.0239.x 15032850

[35] Takahashi Y, Kinoshita T, Shimazaki Ki. Protein phosphorylation and binding of a 14-3-3 protein in vicia guard cells in response to ABA. Plant Cell Physiol 2007; 48: 1182–1191. 17634179 10.1093/pcp/pcm093

[36] Peer WA, Sirichandra C, Davanture M, Turk BE, Zivy M, Valot B, et al The Arabidopsis ABA-activated kinase OST1 phosphorylates the bZIP transcription factor ABF3 and creates a 14-3-3 binding site involved in its turnover. PLoS ONE 2010; 5: e13935 10.1371/journal.pone.0013935 21085673 PMC2978106

[37] Sun X, Luo X, Sun M, Chen C, Ding X, Wang X, et al A *Glycine soja* 14-3-3 protein GsGF14o participates in stomatal and root hair development and drought tolerance in *Arabidopsis thaliana* . Plant Cell Physiol 2014; 55: 99–118. 10.1093/pcp/pct161 24272249

[38] Li X, Dhaubhadel S. Soybean 14-3-3 gene family: identification and molecular characterization. Planta 2010; 233: 569–582. 10.1007/s00425-010-1315-6 21120521

[39] Radwan O, Wu X, Govindarajulu M, Libault M, Neece DJ, Oh MH, et al 14-3-3 proteins SGF14c and SGF14l play critical roles during soybean nodulation. Plant Physiol 2012; 160: 2125–2136. 10.1104/pp.112.207027 23060368 PMC3510136

[40] Li X, Dhaubhadel S. 14-3-3 proteins act as scaffolds for GmMYB62 and GmMYB176 and regulate their intracellular localization in soybean. Plant Signal Behav 2012; 7: 965–968. 10.4161/psb.20940 22836494 PMC3474696

[41] Yi J, Derynck MR, Li X, Telmer P, Marsolais F, Dhaubhadel S. A single-repeat MYB transcription factor, GmMYB176, regulates CHS8 gene expression and affects isoflavonoid biosynthesis in soybean. Plant J 2010; 62:1019–1034. 10.1111/j.1365-313X.2010.04214.x 20345602

[42] DuanMu H, Wang Y, Bai X, Cheng S, Deyholos MK, Wong GK-S, et al Wild soybean roots depend on specific transcription factors and oxidation reduction related genesin response to alkaline stress. Funct Integr Genomics 2015; 15: 651–660. 10.1007/s10142-015-0439-y 25874911

[43] Xu G, Guo C, Shan H, Kong H. Divergence of duplicate genes in exon-intron structure. P Natl Acad Sci USA a 2012; 109: 1187–1192. 10.1073/pnas.1109047109 PMC3268293 22232673

[44] Rogozin IB, Sverdlov AV, Babenko VN, Koonin EV. Analysis of evolution of exon-intron structure of eukaryotic genes. Brief Bioinform 2005; 6: 118–134. 15975222 10.1093/bib/6.2.118

[45] Schmutz J, Cannon SB, Schlueter J, Ma J, Mitros T, Nelson W, et al Genome sequence of the palaeopolyploid soybean. Nature 2010; 463: 178–183. 10.1038/nature08670 20075913

[46] Lee TH, Tang H, Wang X, Paterson AH. PGDD: a database of gene and genome duplication in plants. Nucleic Acids Res 2013; 41: D1152–1158. 10.1093/nar/gks1104 23180799 PMC3531184

[47] Tian F, Wang T, Xie Y, Zhang J, Hu J. Genome-wide identification, classification, and expression analysis of 14-3-3 gene family in Populus. PLoS ONE 2015; 10: e0123225. 25867623 10.1371/journal.pone.0123225 PMC4395111

[48] Nakashima K, Fujita Y, Katsura K, Maruyama K, Narusaka Y, Seki M, et al Transcriptional regulation of ABI3- and ABA-responsive genes including RD29B and RD29A in seeds, germinating embryos, and seedlings of Arabidopsis. Plant Mol Biol 2006; 60: 51–68. 16463099 10.1007/s11103-005-2418-5

[49] Kaplan B, Davydov O, Knight H, Galon Y, Knight MR, Fluhr R, et al Rapid transcriptome changes induced by cytosolic Ca^2+^ transients reveal ABRE-related sequences as Ca^2+^-responsive cis elements in Arabidopsis. Plant Cell 2006; 18: 2733–2748. 16980540 10.1105/tpc.106.042713 PMC1626612

[50] Hattori T, Totsuka M, Hobo T, Kagaya Y, Yamamoto-Toyoda A. Experimentally determined sequence requirement of ACGT-containing abscisic acid response element. Plant Cell Physiol 2002; 43: 136–140. 11828032 10.1093/pcp/pcf014

[51] Narusaka Y, Nakashima K, Shinwari ZK, Sakuma Y, Furihata T, Abe H, et al Interaction between two cis-acting elements, ABRE and DRE, in ABA-dependent expression of Arabidopsis rd29A gene in response to dehydration and high-salinity stresses. Plant J 2003; 34: 137–148. 12694590 10.1046/j.1365-313x.2003.01708.x

[52] Singh A, Jha SK, Bagri J, Pandey GK. ABA inducible rice protein phosphatase 2C confers ABA insensitivity and abiotic stress tolerance in Arabidopsis. PLoS ONE 2015; 10: e0125168 10.1371/journal.pone.0125168 25886365 PMC4401787

[53] Chi JC, Roeper J, Schwarz G, Fischer-Schrader K. Dual binding of 14-3-3 protein regulates Arabidopsis nitrate reductase activity. J Biol Inorg Chem 2015; 20: 277–286. 10.1007/s00775-014-1232-4 25578809

[54] He Y, Wu J, Lv B, Li J, Gao Z, Xu W, et al Involvement of 14-3-3 protein GRF9 in root growth and response under polyethylene glycol-induced water stress. J Exp Bot 2015; 66: 2271–2281. 10.1093/jxb/erv149 25873671 PMC4986726

[55] Chen Q, Kan Q, Wang P, Yu W, Yu Y, Zhao Y, et al Phosphorylation and interaction with the 14-3-3 protein of the plasma membrane H^+^-ATPase are involved in the regulation of magnesium-mediated increases in aluminum-induced citrate exudation in broad bean (*Vicia faba*. L). Plant Cell Physiol 2015; 56: 1144–1153. 10.1093/pcp/pcv038 25745032

[56] Liu Q, Li JG, Ying SH, Wang JJ, Sun WL, Tian CG, et al Unveiling equal importance of two 14-3-3 proteins for morphogenesis, conidiation, stress tolerance and virulence of an insect pathogen. Environ Microbiol 2015; 17: 1444–1462. 10.1111/1462-2920.12634 25315061

[57] Lozano-Duran R, Robatzek S. 14-3-3 proteins in plant-pathogen interactions. Mol Plant Microbe In 2015; 28: 511–518. 10.1094/MPMI-10-14-0322-CR 25584723

[58] Chang IF, Curran A, Woolsey R, Quilici D, Cushman JC, Mittler R, et al Proteomic profiling of tandem affinity purified 14-3-3 protein complexes in Arabidopsis thaliana. Proteomics 2009; 9: 2967–2985. 10.1002/pmic.200800445 19452453 PMC4077669

[59] Wu K, Rooney MF, Ferl RJ. The Arabidopsis 14-3-3 multigene family. Plant Physiol 1997; 114: 1421–1431. 9276953 10.1104/pp.114.4.1421 PMC158435

[60] DeLille JM, Sehnke PC, Ferl RJ. The arabidopsis 14-3-3 family of signaling regulators. Plant Physiol 2001; 126: 35–38. 11351068 10.1104/pp.126.1.35 PMC1540106

[61] Chen F, Li Q, Sun L, He Z. The rice 14-3-3 gene family and its involvement in responses to biotic and abiotic stress. DNA Res 2006; 13: 53–63. 16766513 10.1093/dnares/dsl001

[62] Yao Y, Du Y, Jiang L, Liu JY. Molecular analysis and expression patterns of the 14-3-3 gene family from *Oryza sativa* . J Biochem Mol Biol 2007; 40: 349–357. 17562286 10.5483/bmbrep.2007.40.3.349

[63] Xu WF, Shi WM. Expression profiling of the 14-3-3 gene family in response to salt stress and potassium and iron deficiencies in young tomato (*Solanum lycopersicum*) roots: analysis by real-time RT-PCR. Ann Bot 2006; 98: 965–974. 16943217 10.1093/aob/mcl189 PMC2803592

[64] Sun G, Xie F, Zhang B. Transcriptome-wide identification and stress properties of the 14-3-3 gene family in cotton (*Gossypium hirsutum* L.). Funct Integr Genomic 2011; 11: 627–636. 10.1007/s10142-011-0242-3 21805362

[65] Sehnke PC, Rosenquist M, Alsterfjord M, DeLille J, Sommarin M, Larsson C, et al Evolution and isoform specificity of plant 14-3-3 proteins. Plant Mol Biol 2002; 50: 1011–1018. 12516868 10.1023/a:1021289127519

[66] Yang Y, Yu M, Xu F, Yu Y, Liu C, Li J, et al Identification and expression analysis of the 14-3-3 gene family in the mulberry tree. Plant Mol Biol Rep 2015; In press.

[67] Denison FC, Paul AL, Zupanska AK, Ferl RJ. 14-3-3 proteins in plant physiology. Semin Cell Dev Biol 2011; 22: 720–727. 10.1016/j.semcdb.2011.08.006 21907297

[68] van Kleeff PJ, Jaspert N, Li KW, Rauch S, Oecking C, de Boer AH. Higher order Arabidopsis 14-3-3 mutants show 14-3-3 involvement in primary root growth both under control and abiotic stress conditions. J Exp Bot 2014; 65: 5877–5888. 10.1093/jxb/eru338 25189593 PMC4203132

[69] Chen J, Zhang D, Zhang C, Xia X, Yin W, Tian Q. A Putative PP2C-Encoding Gene Negatively Regulates ABA Signaling in *Populus euphratica* . PLoS ONE 2015; 10: e0139466 10.1371/journal.pone.0139466 26431530 PMC4592019

[70] Zhan G-m, Tong J, Wang H-z, Hua W. Molecular analysis and expression patterns of four 14-3-3 genes from *Brassica napus* L. Agr Sci China 2010; 9: 942–950.

[71] Mayfield JD, Folta KM, Paul AL, Ferl RJ. The 14-3-3 proteins and influence transition to flowering and early phytochrome response. Plant Physiol 2007; 145: 1692–1702. 17951453 10.1104/pp.107.108654 PMC2151679

[72] Tseng TS, Whippo C, Hangarter RP, Briggs WR. The role of a 14-3-3 protein in stomatal opening mediated by PHOT2 in Arabidopsis. Plant Cell 2012; 24: 1114–1126. 10.1105/tpc.111.092130 22408078 PMC3336120

[73] Mayfield JD, Paul AL, Ferl RJ. The 14-3-3 proteins of Arabidopsis regulate root growth and chloroplast development as components of the photosensory system. J Exp Bot 2012; 63: 3061–3070. 10.1093/jxb/ers022 22378945 PMC3350920

[74] Adams E, Diaz C, Hong JP, Shin R. 14-3-3 proteins participate in light signaling through association with PHYTOCHROME INTERACTING FACTORs. Int J Mol Sci 2014; 15: 22801–22814. 10.3390/ijms151222801 25501334 PMC4284738

[75] Mayfield JD, Paul AL, Ferl RJ. The 14-3-3 proteins of Arabidopsis regulate root growth and chloroplast development as components of the photosensory system. J Exp Bot 2012; 63: 3061–3070. 10.1093/jxb/ers022 22378945 PMC3350920

[76] del Viso F, Casaretto JA, Quatrano RS. 14-3-3 Proteins are components of the transcription complex of the ATEM1 promoter in Arabidopsis. Planta 2007; 227: 167–175. 17701425 10.1007/s00425-007-0604-1

[77] Fujita Y, Yoshida T, Yamaguchi-Shinozaki K. Pivotal role of the AREB/ABF-SnRK2 pathway in ABRE-mediated transcription in response to osmotic stress in plants. Physiol Plantarum 2013; 147: 15–27. 10.1111/j.1399-3054.2012.01635.x 22519646

[78] Finkelstein R, Gampala SS, Lynch TJ, Thomas TL, Rock CD. Redundant and distinct functions of the ABA response loci ABA-INSENSITIVE(ABI)5 and ABRE-BINDING FACTOR (ABF)3. Plant Mol Biol 2005; 59: 253–267. 16247556 10.1007/s11103-005-8767-2

[79] Li W, Yadeta KA, Elmore JM, Coaker G. The *Pseudomonas syringae* effector HopQ1 promotes bacterial virulence and interacts with tomato 14-3-3 proteins in a phosphorylation-dependent manner. Plant Physiol 2013; 161: 2062–2074. 10.1104/pp.112.211748 23417089 PMC3613476

[80] Giska F, Lichocka M, Piechocki M, Dadlez M, Schmelzer E, Hennig J, et al Phosphorylation of HopQ1, a type III effector from *Pseudomonas syringae*, creates a binding site for host 14-3-3 proteins. Plant Physiol 2013; 161: 2049–2061. 10.1104/pp.112.209023 23396834 PMC3613475

[81] de Boer AH, van Kleeff PJ, Gao J. Plant 14-3-3 proteins as spiders in a web of phosphorylation. Protoplasma 2013; 250: 425–440. 10.1007/s00709-012-0437-z 22926776

[82] Ichimura T, Taoka M, Hozumi Y, Goto K, Tokumitsu H. 14-3-3 Proteins directly regulate Ca^2+^/calmodulin-dependent protein kinase kinase α through phosphorylation-dependent multisite binding. FEBS Lett 2008; 582: 661–665. 10.1016/j.febslet.2008.01.037 18242179

[83] Cai S, Jiang G, Ye N, Chu Z, Xu X, Zhang J, et al A key ABA catabolic gene, *OsABA8ox3*, is involved in drought stress resistance in rice. PLoS ONE 2015; 10: e0116646 10.1371/journal.pone.0116646 25647508 PMC4315402

[84] Xu DB, Chen M, Ma YN, Xu ZS, Li LC, Chen YF, et al A G-protein beta subunit, AGB1, negatively regulates the ABA response and drought tolerance by down-regulating AtMPK6-related pathway in Arabidopsis. PLoS ONE 2015; 10: e0116385 10.1371/journal.pone.0116385 25635681 PMC4312036

[85] Ren X, Chen Z, Liu Y, Zhang H, Zhang M, Liu Q, et al ABO3, a WRKY transcription factor, mediates plant responses to abscisic acid and drought tolerance in Arabidopsis. Plant J 2010; 63: 417–429. 10.1111/j.1365-313X.2010.04248.x 20487379 PMC3117930

[86] Sridha S, Wu K. Identification of AtHD2C as a novel regulator of abscisic acid responses in Arabidopsis. Plant J 2006; 46: 124–133. 16553900 10.1111/j.1365-313X.2006.02678.x

[87] Yang CY, Chen YC, Jauh GY, Wang CS. A Lily ASR protein involves abscisic acid signaling and confers drought and salt resistance in Arabidopsis. Plant Physiol 2005; 139: 836–846. 16169963 10.1104/pp.105.065458 PMC1255999

[88] Wang J, Zheng R, Bai S, Gao X, Liu M, Yan W. Mongolian almond (*Prunus mongolica* Maxim): the morpho-physiological, biochemical and transcriptomic response to drought stress. PLoS ONE 2015; 10: e0124442 10.1371/journal.pone.0124442 25893685 PMC4404049

[89] Benesova M, Hola D, Fischer L, Jedelsky PL, Hnilicka F, Wilhelmova N, et al The physiology and proteomics of drought tolerance in maize: early stomatal closure as a cause of lower tolerance to short-term dehydration? PLoS ONE 2012; 7: e38017 10.1371/journal.pone.0038017 22719860 PMC3374823

[90] Hu B, Jin J, Guo AY, Zhang H, Luo J, Gao G. GSDS 2.0: an upgraded gene feature visualization server. Bioinformatics 2015; 31: 1296–1297. 10.1093/bioinformatics/btu817 25504850 PMC4393523

[91] Yang J, Yan R, Roy A, Xu D, Poisson J, Zhang Y. The I-TASSER Suite: protein structure and function prediction. Nat methods 2015; 12: 7–8. 10.1038/nmeth.3213 25549265 PMC4428668

[92] Higo K, Ugawa Y, Iwamoto M, Korenaga T. Plant cis-acting regulatory DNA elements (PLACE) database: 1999. Nucleic Acids Res 1999; 27: 297–300. 9847208 10.1093/nar/27.1.297 PMC148163

[93] Yang L, Ji W, Gao P, Li Y, Cai H, Bai X, et al GsAPK, an ABA-activated and calcium-independent SnRK2-type kinase from G. soja, mediates the regulation of plant tolerance to salinity and ABA stress. PLoS ONE 2012; 7: e33838 10.1371/journal.pone.0033838 22439004 PMC3306294

[94] Tocquin P, Corbesier L, Havelange A, Pieltain A, Kurtem E, Bernier G, et al A novel high efficiency, low maintenance, hydroponic system for synchronous growth and flowering of *Arabidopsis thaliana* . BMC Plant Biol 2003; 3: 2 12556248 10.1186/1471-2229-3-2 PMC150571

[95] Jefferson RA, Kavanagh TA, Bevan MW. GUS fusions: beta-glucuronidase as a sensitive and versatile gene fusion marker in higher plants. EMBO J 1987; 6: 3901–3907. 3327686 10.1002/j.1460-2075.1987.tb02730.x PMC553867

[96] Bates L, Waldren R, Teare I. Rapid determination of free proline for water-stress studies. Plant and Soil 1973; 39: 3.

