# Supplementary figures and images for: A 14-3-3 Family Protein from Wild Soybean (Glycine Soja) Regulates ABA Sensitivity in Arabidopsis
Source: PLoS One. 2015 Dec 30;10(12):e0146163. doi: 10.1371/journal.pone.0146163 (PMC4696740; doi:10.1371/journal.pone.0146163)

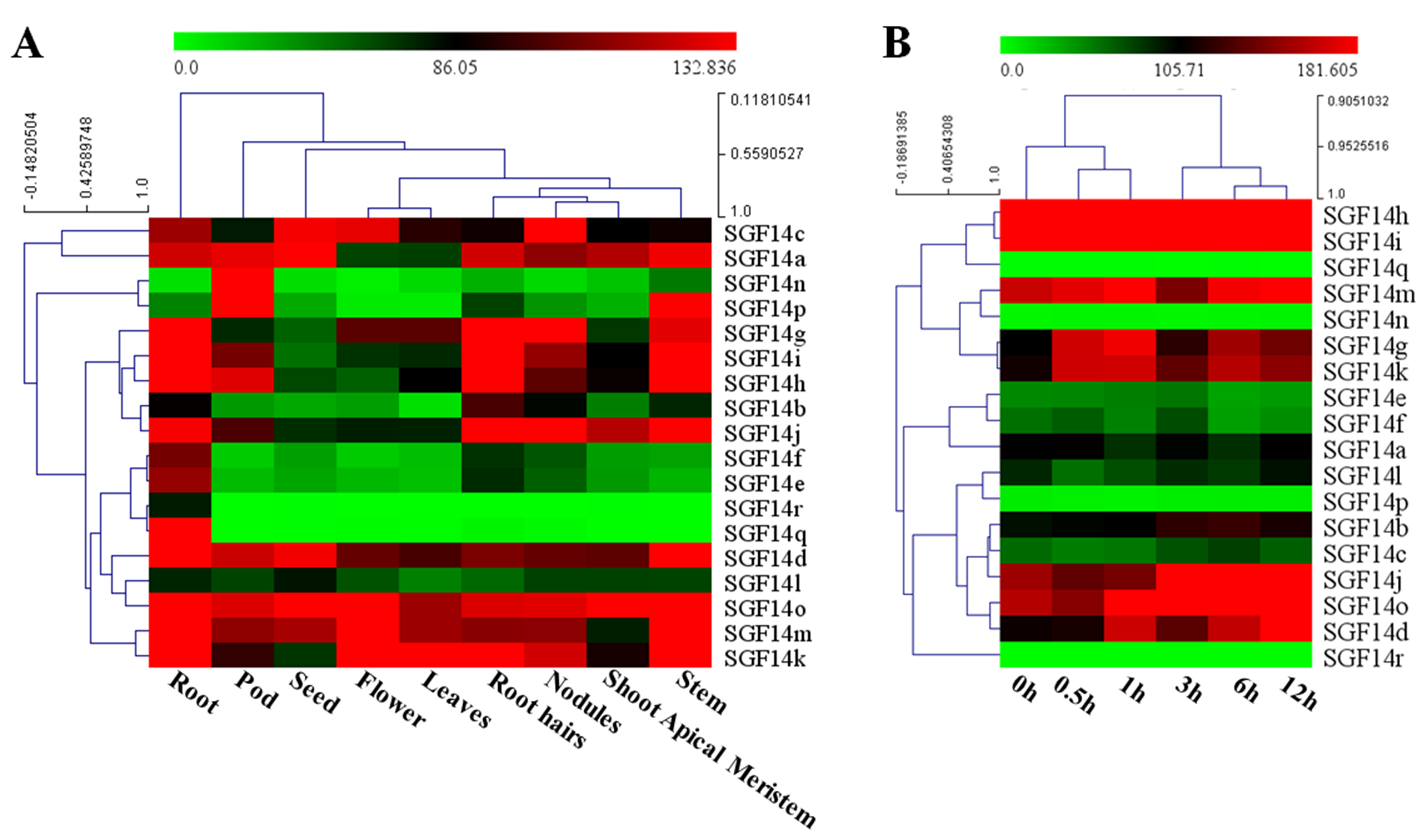

Supplement: S1 Fig — (A) Expression profiles of soybean 14-3-3s in different tissues. (B) Expression profiles of soybean 14-3-3s in response to alkaline stress (50 mM NaHCO3, pH 8.5) based on the RNA-seq data. (TIF) [file pone.0146163.s001.tif]

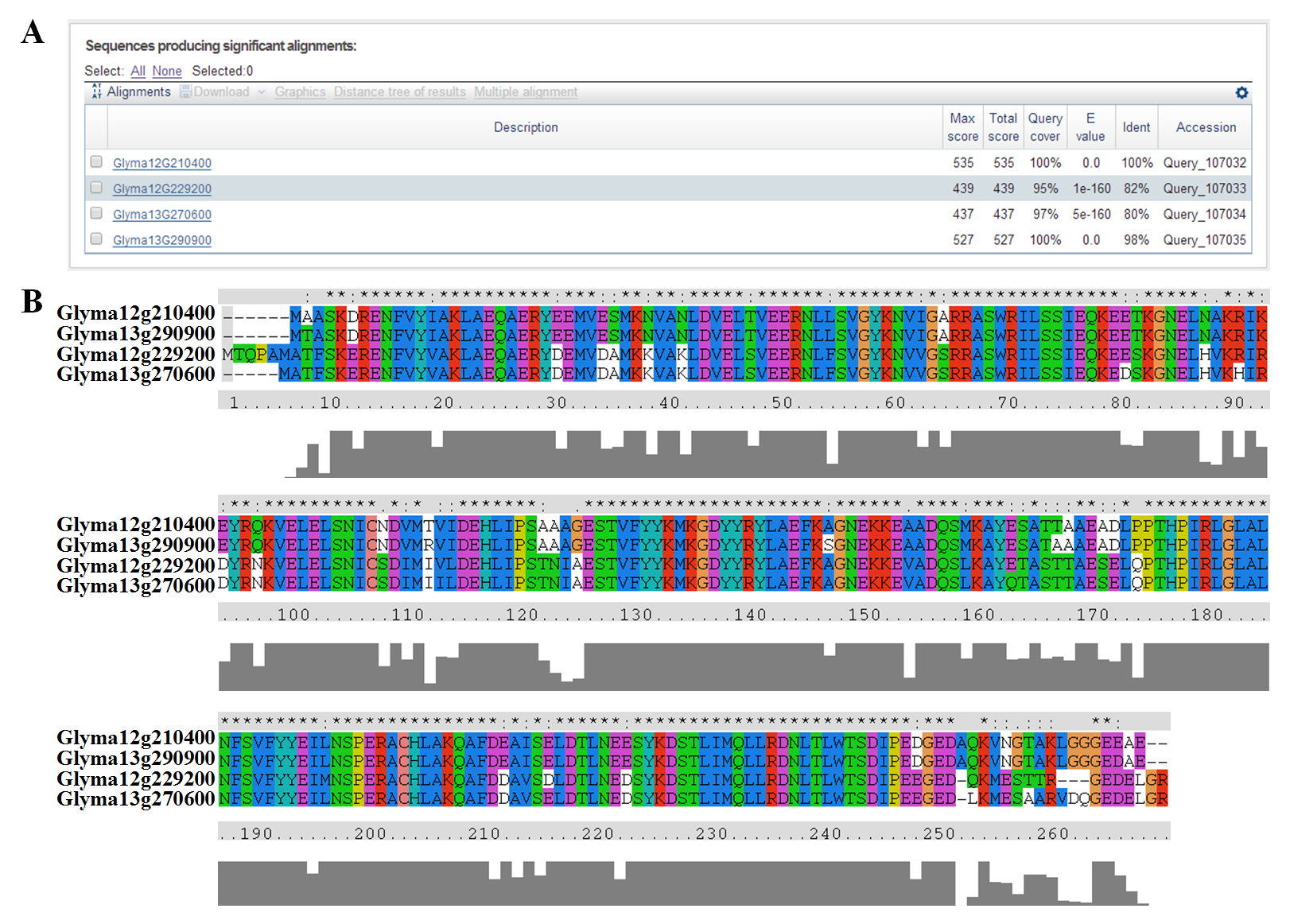

Supplement: S2 Fig — (A) Sequence identity among the four group I 14-3-3 proteins. (B) Multiple sequence alignment of the four group I 14-3-3 proteins. (TIF) [file pone.0146163.s002.tif]

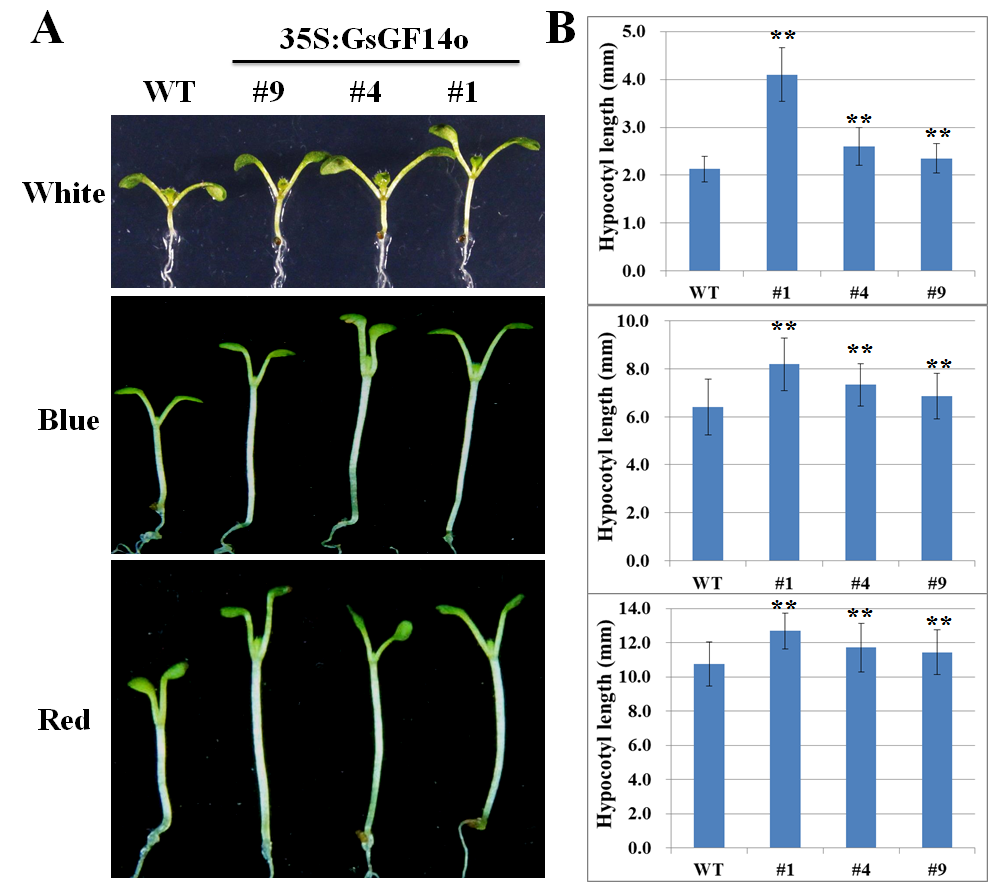

Supplement: S3 Fig — (A) Representative photos to show the hypocotyls from WT and GsGF14o OX seedlings under white, blue and red light. (B) Comparison of the hypocotyl length of WT and OX lines. (TIF) [file pone.0146163.s003.tif]
